# Enteric fever cluster identification in South Africa using genomic surveillance of *Salmonella enterica* serovar Typhi

**DOI:** 10.1099/mgen.0.001044

**Published:** 2023-06-20

**Authors:** Anthony Marius Smith, Linda Kathleen Erasmus, Nomsa Pauline Tau, Shannon Lucrecia Smouse, Hlengiwe Mimmy Ngomane, Bolele Disenyeng, Andrew Whitelaw, Charlene Ann Lawrence, Phuti Sekwadi, Juno Thomas

**Affiliations:** ^1^​ Centre for Enteric Diseases, National Institute for Communicable Diseases, Division of the National Health Laboratory Service, Johannesburg, South Africa; ^2^​ Department of Medical Microbiology, Faculty of Health Sciences, University of Pretoria, Pretoria, South Africa; ^3^​ Department of Pathology, Faculty of Medicine and Health Sciences, Stellenbosch University, Cape Town, South Africa; ^4^​ National Health Laboratory Service, Tygerberg Hospital, Cape Town, South Africa; ^5^​ Communicable Disease Control, Service Priorities Coordination, Department of Health, Cape Town, South Africa

**Keywords:** *Salmonella *Typhi, enteric fever, whole-genome sequencing, genomic, WGS, surveillance, cluster, outbreak, South Africa, Africa

## Abstract

The National Institute for Communicable Diseases in South Africa participates in national laboratory-based surveillance for human isolates of *Salmonella* species. Laboratory analysis includes whole-genome sequencing (WGS) of isolates. We report on WGS-based surveillance of *Salmonella enterica* serovar Typhi (* Salmonella* Typhi) in South Africa from 2020 through 2021. We describe how WGS analysis identified clusters of enteric fever in the Western Cape Province of South Africa and describe the epidemiological investigations associated with these clusters. A total of 206 *Salmonella* Typhi isolates were received for analysis. Genomic DNA was isolated from bacteria and WGS was performed using Illumina NextSeq technology. WGS data were investigated using multiple bioinformatics tools, including those available at the Centre for Genomic Epidemiology, EnteroBase and Pathogenwatch. Core-genome multilocus sequence typing was used to investigate the phylogeny of isolates and identify clusters. Three major clusters of enteric fever were identified in the Western Cape Province; cluster one (*n*=11 isolates), cluster two (*n*=13 isolates), and cluster three (*n*=14 isolates). To date, no likely source has been identified for any of the clusters. All isolates associated with the clusters, showed the same genotype (4.3.1.1.EA1) and resistome (antimicrobial resistance genes: *bla*_TEM-1B_, *catA1*, *sul1*, *sul2*, *dfrA7*). The implementation of genomic surveillance of *Salmonella* Typhi in South Africa has enabled rapid detection of clusters indicative of possible outbreaks. Cluster identification allows for targeted epidemiological investigations and a timely, coordinated public health response.

## Data Summary

Supplementary material can be found in the online version of this article. These supplementary material include: (1) NICD Enteric fever (typhoid and paratyphoid fever): Recommendations for diagnosis, management and public health response [1], Enteric fever case investigation form [2], Metadata for *Salmonella* Typhi isolates, Western Cape Province, South Africa, 2020–2021.

All sequencing data were uploaded to the public EnteroBase platform (http://enterobase.warwick.ac.uk/species/index/senterica) and so are freely available to access at the EnteroBase platform. In addition, sequencing data are deposited in the European Nucleotide Archive under the project accession number PRJEB39546.

**Impact Statement**

*
Salmonella* Typhi, the causative bacterial agent of typhoid fever, remains endemic in many African countries. Active surveillance for typhoid fever and rapid identification/characterization of the causative agent is important to initiate appropriate public heath responses, identify the source of the agent and control spread of the disease. Whole-genome sequencing (WGS) analysis of bacterial pathogens is the ultimate laboratory methodology. WGS data allows for full characterization of bacterial isolates, and importantly can be used to investigate the genetic relatedness of isolates, in order to investigate the source and spread of isolates. WGS is revolutionizing and transforming communicable diseases surveillance, allowing for more targeted investigations. WGS data can be used to identify clusters of cases in real-time and complement epidemiological investigation of outbreaks. Cluster identification allows for targeted epidemiological investigations and a timely, coordinated public health response. The National Institute for Communicable Diseases (NICD), South Africa participates in national laboratory-based surveillance for human isolates of *Salmonella* species. Laboratory analysis includes WGS of isolates. We report on WGS-based surveillance of *Salmonella* Typhi in South Africa from 2020 through 2021. We describe how WGS analysis identified clusters of enteric fever in the Western Cape Province of South Africa and describe the epidemiological investigations associated with these clusters.

## Introduction

*Salmonella enterica* remains a major cause of human disease worldwide [1, 3]. *Salmonella enterica* serovar Typhi (* Salmonella* Typhi), the causative agent of typhoid fever, is of particular concern. The World Health Organization estimates the global typhoid fever disease burden at 11–20 million cases yearly, resulting in about 128000–161000 deaths per year [2]. Sub-Saharan Africa bears a substantial burden of disease, with most countries in this region reported to be endemic for typhoid fever [4–7].

In South Africa, the incidence rates of typhoid fever have decreased markedly over the last decades, but the disease remains endemic [8]. Over the past 15 years, an average of 97 laboratory-confirmed cases of enteric fever were reported per year [9, 10]. Outbreaks of typhoid fever have been documented in South Africa; the most recent large outbreak having occurred in 2005, in Delmas, Mpumalanga Province, where over 2900 cases were reported [11–13]. Typhoid fever remains a public health problem in the country, and it is important that a robust surveillance system is in place to initiate a timely public health response to every case, and to rapidly identify possible outbreaks. Enteric fever is a notifiable medical condition in South Africa, meaning that all laboratory-confirmed cases must be reported to the National Department of Health. This notification system is complemented by appropriate laboratory characterization of *Salmonella* Typhi isolates, including molecular subtyping of isolates to investigate their genetic relatedness [11, 14, 15]. The Centre for Enteric Diseases (CED) at the National Institute for Communicable Diseases (NICD) has recently integrated whole-genome sequencing (WGS) analysis of all *Salmonella* Typhi isolates into its surveillance system. This step to WGS was necessary to keep abreast with advances in microbiological methodologies and follow global trends in that many reference laboratories in global public institutions have transitioned to WGS as their primary methodology for molecular subtyping and characterization of bacterial pathogens [16–19]. WGS is revolutionizing and transforming communicable diseases surveillance, allowing for more targeted investigations. WGS data can be used to identify clusters of cases in real-time and complement epidemiological investigation of outbreaks.

We report on the use of WGS for real-time genomic surveillance of *Salmonella* Typhi in South Africa from 2020 through 2021. We describe how genomic analysis led to the identification of multiple clusters of enteric fever in the Western Cape Province and describe the epidemiological findings associated with the investigation of these clusters.

## Methods

### Surveillance for *Salmonella* Typhi in South Africa

The NICD is the national public health institute of South Africa, providing reference microbiology, virology, epidemiology, surveillance and public health research and training to support the government’s response to communicable disease threats. The CED at the NICD participates in national laboratory-based surveillance for human isolates of *Salmonella* species. All clinical isolates of *Salmonella* species are collected by voluntary submissions from >200 clinical microbiology laboratories across the country [9, 10]. Following a *Salmonella* species identification at a clinical microbiology laboratory, the isolate is received at the CED within 1 week. The CED then immediately commences with phenotypic characterization and WGS analysis (as described below), which is completed within 2–3 weeks. Notification of *Salmonella* Typhi cases through a notifiable medical conditions surveillance system enables audit and follow up of isolates that were not received. Isolates are accompanied by basic metadata (demographic data) including patient details, place of residence, and date of specimen collection.

### Epidemiologic case investigation

Enteric fever is a notifiable disease in South Africa, with standard guidelines for case investigation (Supplementary material, available in the online version of this article) [20]. Health authorities received notification directly through the national notifiable medical conditions surveillance system within 24 h of laboratory confirmation and initiated the case investigation. Clinical and demographic details and underlying medical conditions were ascertained through patient interviews or abstracted from medical records with the use of a standardized case-investigation form (Supplementary material). Case patients (or next of kin or caregivers in cases in which the patient was a child, had died, or was too ill to respond) were interviewed to ascertain risk factors for infection with *Salmonella* Typhi, including travel, occupation, sources of water used in the home, exposure to water sources outside the home, sanitation and hygiene in the home, and sources of food consumed in the month prior to onset of illness. Faecal samples (stool or rectal swabs) were collected from household contacts where possible. Health authorities conducted an environmental health assessment at the patient’s home (and when indicated, place of work) which informed further investigation of possible foodborne or waterborne sources of infection. This may have included sampling of food or water. Water samples were collected by environmental health authorities. At least one litre of water was collected per sampling site and stored at 2–8 °C during transport to the laboratory, and tested according to ISO (International Organization for Standardization) standard 19 250 for the detection of *Salmonella *. Additionally, when clusters were identified on genomic analysis all case investigations were reviewed and additional investigations performed.

### Phenotypic characterization of bacteria

The CED received *Salmonella* Typhi on Dorset-Egg transport media [Diagnostic Media Products (DMP), National Health Laboratory Service, Johannesburg, South Africa] and sub-cultured onto 5 % Blood Agar (DMP), to check for viability and purity. Cultures were identified using standard phenotypic microbiological identification and serotyping techniques, briefly described as follows. As required, bacterial colonies were identified using the VITEK-2 COMPACT 15 automated microbial identification system (bioMérieux, Marcy-l'Étoile, France). Serotyping was performed according to the White-Kauffmann-Le Minor Scheme [21]. Antimicrobial (ampicillin, chloramphenicol, ciprofloxacin, ceftriaxone, azithromycin) susceptibility testing was performed using the Etest method (bioMérieux). Interpretation of antimicrobial susceptibility data was done in accordance with the Clinical and Laboratory Standards Institute (CLSI) [22].

### Whole-genome sequencing (WGS) of bacteria

Genomic DNA was isolated from bacteria using an Invitrogen PureLink Microbiome DNA Purification Kit (Invitrogen, Waltham, Massachusetts, USA). WGS was performed using Illumina NextSeq (Illumina, San Diego, California, USA) next generation sequencing technology, with DNA libraries prepared using a Nextera DNA Flex Library Preparation Kit (Illumina), followed by 2×150 bp paired-end sequencing runs with ~80 times coverage. All these methodologies associated with WGS, were performed using the protocols and standard operating procedures as described by the manufacturers of the kits and equipment.

### Analysis of WGS data

Illumina paired-end reads were analysed using the JEKESA bioinformatics pipeline version 1.0 (https://github.com/stanikae/jekesa), which incorporates multiple bioinformatics analysis tools, briefly described as follows. All underlying tools were set to default options, unless otherwise stated below. Quality control and read filtering of raw paired-end reads were performed using FastQC version 0.11.9 (https://www.bioinformatics.babraham.ac.uk/projects/fastqc/) and TrimGalore version 0.6.2 (https://github.com/FelixKrueger/TrimGalore) set to a minimum Phred quality score of 30 and minimum read length of 50 bp. Species identification and closest reference detection were performed using BactInspector version 0.1.3 (https://gitlab.com/antunderwood/bactinspector). Contamination checks were performed using ConFindr version 0.7.4 [23] and Kraken2 version 2.0.8-beta [24]. *De novo* assembly was performed using SKESA version 2.3.0 [25], and the assemblies were optimized using Shovill version 1.1.0 (https://github.com/tseemann/shovill) with depth set to 100 and minimum contig length set to 200. Assembly metrics were assessed using QUAST version 5.0.2 [26]. The assembled genomes were further investigated using the following tools. Multilocus sequence typing (MLST) was performed using mlst version 2.19.0 (https://github.com/tseemann/mlst), based on PubMLST typing schemes (https://pubmlst.org/). Detection of antimicrobial resistance determinants were performed using ResFinder version 4.1 (http://www.genomicepidemiology.org/services/) [27]. *Salmonella* serovar prediction was performed using SeqSero2 version 1.1.0 (http://denglab.info/SeqSero2) [28] and SISTR version 1.1.2 (https://github.com/phac-nml/sistr_cmd) [29]. Plasmid DNA presence was investigated using PlasmidFinder version 2.1 (http://www.genomicepidemiology.org/services/). Information regarding *Salmonella* Typhi genotype [as called by single nucleotide polymorphism (SNP) analysis] was determined using the GenoTyphi Scheme [30] at the Pathogenwatch platform (https://pathogen.watch/).

Core-genome MLST (cgMLST) was used to investigate the phylogeny and genetic relatedness of isolates. This was made possible using the cgMLST scheme available at the EnteroBase platform (http://enterobase.warwick.ac.uk/species/index/senterica). Raw sequencing data (FastQ files for paired-end reads) were uploaded and investigated at EnteroBase using the cgMLST tool (using the ‘cgMLST V2 +HierCC V1’ scheme which incorporates analysis at 3002 genes). Further information regarding the operational tools of EnteroBase and tutorials on how to use the tools, can be found at the following URL links: https://enterobase.readthedocs.io/en/latest/, https://bitbucket.org/enterobase/enterobase-web/wiki/GrapeTree, https://bitbucket.org/enterobase/enterobase-web/wiki/Tutorials, https://enterobase.readthedocs.io/en/latest/enterobase-tutorials/tutorials.html. In addition, EnteroBase tools have previously been described by Zhou and coworkers [31]. The phylogeny and genetic relatedness of isolates was depicted using a GrapeTree-generated minimum spanning tree using the ‘MSTree V2’ algorithm, tools which are all integrated within EnteroBase [32]. Once the GrapeTree was produced, the settings/operators of the tool were set to ‘collapse braches’ at a value of ‘5’, which resulted in isolates showing ≤5 allelic differences to collapse together into a ‘cluster’. This was how a cluster of isolates was created and visualized. Our cluster definition was ≥3 isolates showing ≤5 allelic differences, as obtained by the above actions, following cgMLST analysis and generation of a GrapeTree. It is important to note that clusters were not defined by the cgMLST hierarchical cluster tool, i.e. not defined at the hierarchical cluster level 5 (HC5). In addition, clusters generated by our above described actions (visualization on a GrapeTree) are not always directly comparable to cluster number generated by HC5.

We further investigated genetic variation within clusters using a single nucleotide polymorphism (SNP) analysis of isolates using CSI Phylogeny version 1.4 (https://cge.food.dtu.dk/services/CSIPhylogeny/) [33].

## Results

**Fig. 1.**
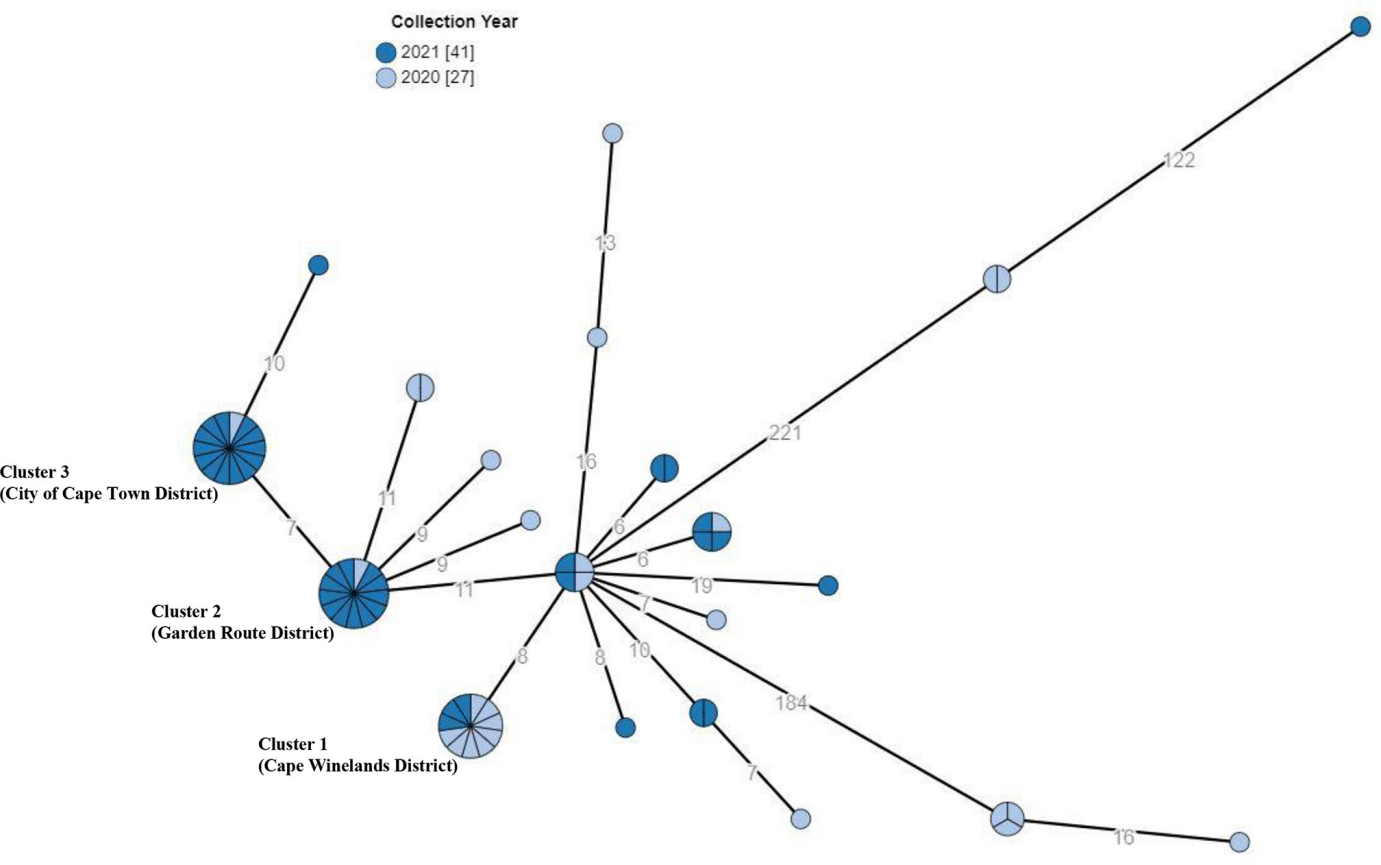
Minimum spanning tree drawn using cgMLST data from *Salmonella* Typhi isolates (n=68) sourced from the Western Cape Province of South Africa, 2020-2021. The circular nodes represent isolate(s). Isolates showing ≤5 allelic differences, are collapsed together into a single circular node. The larger the circular node, the more isolates which are reflected. The number of segments within a circular node are indicative of the number of isolates. The number values between adjacent nodes indicate the number of allele differences between connecting nodes (isolates). Three major clusters of isolates are indicated.

**Fig. 2.**
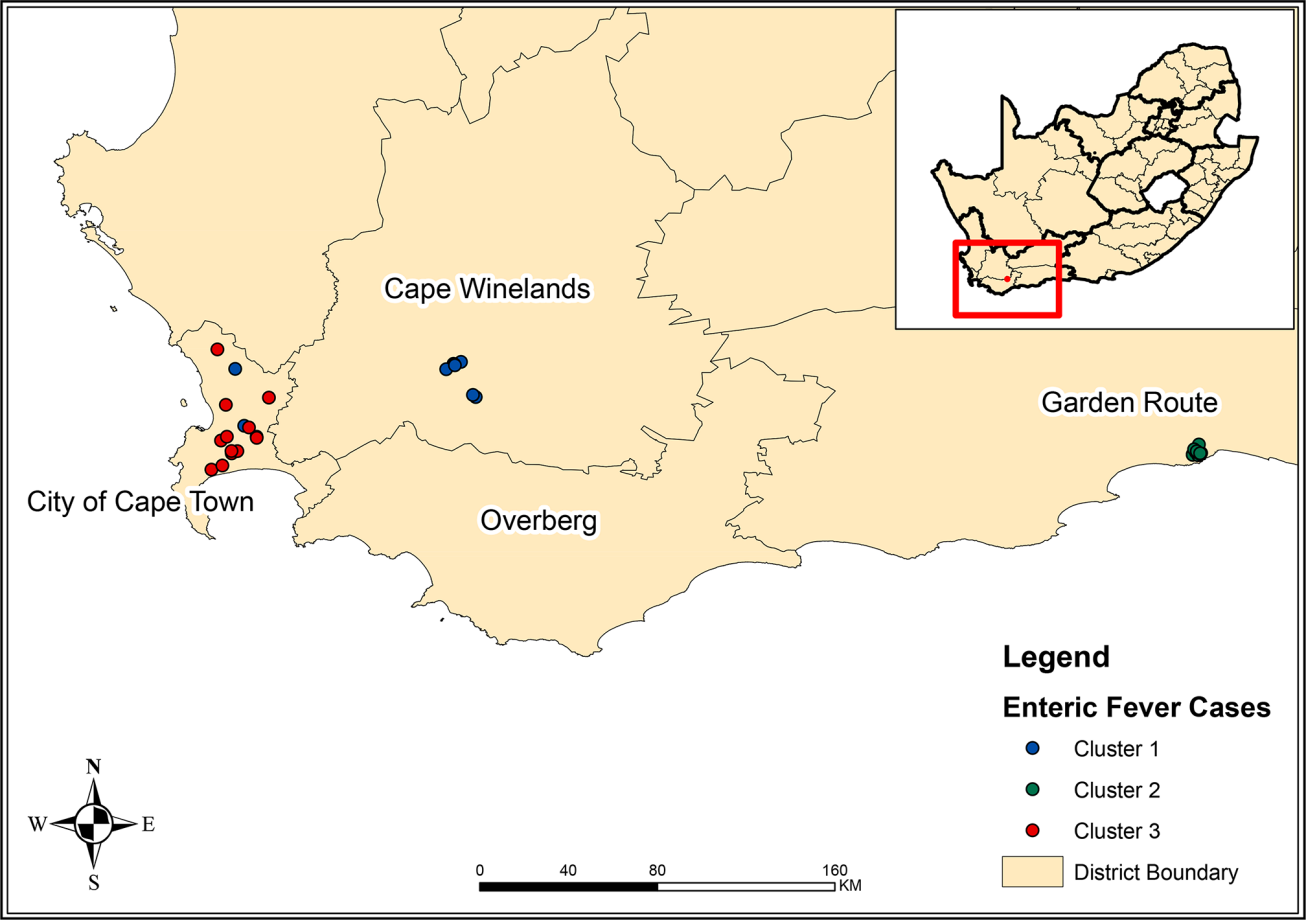
Map of the Western Cape Province of South Africa showing the location of three major *Salmonella* Typhi clusters identified from 2020 through 2021.

**Fig. 3.**
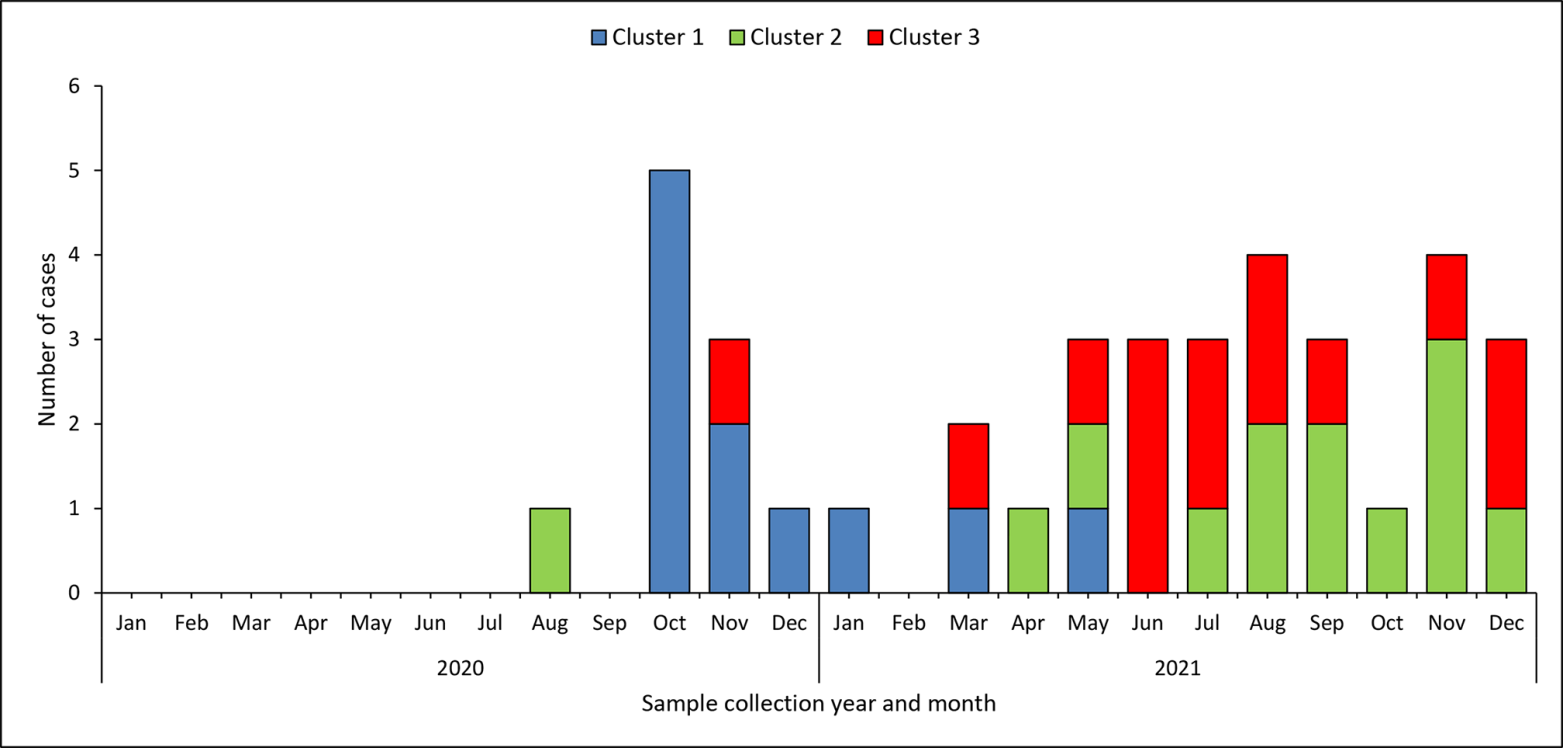
Epidemiological curve showing the month of sample collection in 2020–2021, for all isolates associated with three major Salmonella Typhi clusters identified in the Western Cape Province of South Africa.

We performed an analysis of WGS and cgMLST data of *Salmonella* Typhi isolates (*n*=68) from the Western Cape Province of South Africa for 2020 through 2021. As per our cluster definition (described in the Methodology section), we identified three major clusters of isolates (≥11 isolates per cluster) and three minor clusters of isolates (≤4 isolates per cluster) (Fig. 1). The minor clusters were not further investigated due to human resource constraints. We simply do not have the resources to perform extended epidemiological investigations on every case. So, all our efforts were directed to focus on the three major clusters. These major clusters were identified in three districts of the Western Cape Province (Fig. 2). Cluster one was associated with Cape Winelands District (Breede Valley Sub-District) (*n*=11 isolates), cluster two with Garden Route District (George Sub-District) (*n*=13 isolates), and cluster three with the City of Cape Town District (*n*=14 isolates) (Figs 1–3) (Supplementary material). Epidemiological investigations were conducted on these three major clusters.

Cluster one comprised 11 cases, identified from October 2020 through May 2021 (Figs 1–3). Ten cases were diagnosed on blood culture and one on stool culture. The majority of case patients (8/11; 73 %) were 15 years of age or younger, and males predominated (8/11; 73 %). Nine of the eleven case patients (9/11; 82 %) lived in Cape Winelands District, and two lived in City of Cape Town District but reported recent travel to Cape Winelands District. Four case-patients reported swimming in a river-water reservoir system in Cape Winelands District, so contamination of this surface water system was initially suspected to be the source of infection. However, *Salmonella* species was not isolated from multiple water samples collected from the river and water reservoir, and since most cases reported no exposure to this water system, it was concluded that this river-water reservoir system was unlikely to be a source of infection.

Cluster two comprised 13 cases. The first case was identified in August 2020, followed by an 8 month hiatus, after which 12 cases were identified from April 2021 through to December 2021 (Figs 1–3). Nine cases were diagnosed on blood culture and four on stool culture. The majority of case patients (11/13; 85 %) were 15 years of age or younger, and males predominated (8/13; 62 %). All case patients lived in the same George Sub-District; 8/13 lived within the same township and 2/8 lived in the same street. The case-patients lived in residential areas with inadequate sanitation and unhygienic conditions, including illegally dumped waste, pools of stagnant water from overflowing storm water drains, overflowing sewage works and man-made urinal stalls. Several samples of stagnant water pools and river water were tested, but *Salmonella* species was not detected. Case-patients of school-going age attended several different schools in the district, but through a school feeding programme all scholars in the district were served daily meals prepared by a single service provider. Attempts to investigate the service provider were unsuccessful and stool samples could not be collected from food handlers, so this possible common source of infection could not be confirmed or excluded.

Cluster three comprised 14 cases. The first case was identified in November 2020, followed by a 4 month hiatus, after which 13 cases were identified from March 2021 through to December 2021 (Figs 1–3). All cases were diagnosed on blood culture. Among case patients, males predominated (9/14; 64 %); the age of patients ranged from 7 to 54 years (median age of 21 years), with only five cases (36 %; 5/14) aged 15 years or younger. The cases were detected in the City of Cape Town District; case-patients resided in multiple townships in a large geographical area and were diagnosed at several public hospitals in the district. Investigations revealed no obvious epidemiological links between cases, and nothing to suggest exposure to a common source of infection. The widespread distribution of cases across the district could not inform targeted investigation with environmental sampling.

Epidemiological investigation of these clusters resulted in public health responses and activities, which included, the detection and treatment of asymptomatic carriers among the close contacts of cases, health communication to affected communities, and more frequent environmental sampling to monitor water quality.

All isolates associated with the three clusters showed similar phenotypic and genotypic properties. Genotypic properties for isolates are summarized in Table 1 and are briefly described as follows. All isolates were ST1, all showed genotype 4.3.1.1.EA1, and all showed a multidrug resistance (MDR) genotype with presence of multiple antimicrobial resistance (AMR) genes (*bla*_TEM-1B_, *catA1*, *sul1*, *sul2*, and *dfrA7*). Phenotypically, the isolates all showed resistance to ampicillin (MIC, 256 µg ml^−1^) and chloramphenicol (MIC, 256 µg ml^−1^); while all showed susceptibility to ciprofloxacin (MIC, ≤0.06 µg ml^−1^), ceftriaxone (MIC, ≤1 µg ml^−1^), and azithromycin (MIC, ≤16 µg ml^−1^).

**Table 1.** Genotypic properties of *Salmonella* Typhi isolates associated with three major clusters identified in the Western Cape Province of South Africa, 2020–2021

Cluster no.	Mlst*	cgMLST HC2 no.†	cgMLST HC5 no.†	cgMLST HC50 no.†	Genotype‡	Presence of acquired AMR genes	Mutations in the QRDRs of gyrA, gyrB, parC and parE	Plasmid presence
1	1	26 478	26 478	202	4.3.1.1.EA1	*bla*_TEM-1B_, *catA1*, *sul1*, *sul2*, *dfrA7*	None	IncQ1
2	1	256 191 (*n*=12), 295 929 (*n*=1)	202	202	4.3.1.1.EA1	*bla*_TEM-1B_, *catA1*, *sul1*, *sul2*, *dfrA7*	None	IncQ1
3	1	12 503 (*n*=13), 221 038 (*n*=1)	202	202	4.3.1.1.EA1	*bla*_TEM-1B_, *catA1*, *sul1*, *sul2*, *dfrA7*	None	IncQ1

*As per assignment at PubMLST (https://pubmlst.org/).

†As per assignment at EnteroBase (http://enterobase.warwick.ac.uk/species/index/senterica).

‡As per assignment at Pathogenwatch (https://pathogen.watch/).

cgMLST, core-genome MLST.; MLST, multilocus sequence typing; QRDRs, quinolone resistance-determining regions.

## Discussion

In recent years, next-generation sequencing (NGS) technology has rapidly advanced, resulting in shorter turnaround-times to WGS results and higher accuracy of sequencing data [19]. In parallel, costs of NGS have also decreased dramatically, making the technology more affordable and cost-effective for use in public health laboratories [18]. As a result, reference laboratories in many public institutions across the world have transitioned to WGS as the primary methodology for assessing relatedness of bacterial isolates for surveillance activities, cluster searches and outbreak investigations [16, 17]. The NICD is a national public health institute in South Africa and houses a Sequencing Core Facility which is well-established, well-equipped and well-resourced to perform NGS in all applications of the technology. Many reference laboratories at the NICD have already implemented NGS and WGS for surveillance, outbreak investigation and research purposes. In particular, the CED at the NICD has implemented routine WGS analysis of all outbreak-prone enteric bacterial pathogens (including *Salmonella* species) into its laboratory-based surveillance system [34–36]. Our turnaround time, from receipt of a culture at the CED laboratory to completion of analysis of WGS data, is 2–3 weeks.

In South Africa, over the past 15 years, an average of 97 laboratory-confirmed cases of enteric fever were reported per year (range 66–140) [37]. In 2020 and 2021, 89 and 134 cases were reported per year respectively. Of the 223 cases in these 2 years, *Salmonella* Typhi isolates were received for 92 % (206/223). Although the national number of cases of enteric fever in 2021 was within the expected range, at a provincial level there was a striking increase in cases in the Western Cape Province. From 2003 through 2020 an average of 21 cases (range 8–40) were reported annually in this province; in 2021, a total of 52 cases were reported, which was the highest number of annual cases since 2003 [37]. This increase in cases in Western Cape Province was from three districts (Cape Winelands, Garden Route and City of Cape Town) (Fig. 2). Using WGS analysis of *Salmonella* Typhi isolates, we identified three major enteric fever clusters in the Western Cape Province of South Africa from 2020 through 2021. A cluster indicates a possible common source of infection and could herald a larger outbreak. The relevant health departments were notified of the clusters, and a multi-stakeholder team was convened to investigate them. Unfortunately, no confirmed source(s) of infection were identified for any of the clusters. Contamination of municipal potable water was extremely unlikely to be the source of infection in any of the clusters, due to the demographics of the cases (including age profiles, places of residence, source(s) of drinking water and access to improved sanitation), and culture-negative environmental sampling. In particular, the size of the clusters (fewer than 15 cases in each), and the slow accumulation of cases over several months provided further evidence to exclude contamination of municipal potable water as the primary source of infection. It is very likely that there are complex chains of transmission within the respective communities, mostly due to the presence of unrecognised cases and carriers who serve as reservoirs of infection and lead to ongoing transmission. This makes it challenging to investigate and pinpoint source(s) and reservoir(s) of infection within the communities. The same difficulty in identifying the source(s) of genetically highly related *Salmonella* Typhi strains has been reported elsewhere [38].

All isolates associated with our three major enteric fever clusters showed genotype 4.3.1.1.EA1 (the H58 haplotype strain) [30, 39]. The cgMLST hierarchical cluster tool at the EnteroBase platform was used to further interrogate our isolates (Table 1). At HC50, where isolates are clustered at 50 allele differences; all cluster isolates showed HC50 : 202. The EnteroBase assignment HC50 : 202 is known to be indicative of the H58 haplotype, so all was in agreement with the SNP-defined assignment of genotype 4.3.1.1.EA1. Interrogation at the HC5 and HC2 levels provided more sensitive discrimination of our isolates. For cluster one isolates, at the HC2 level, all were assigned as HC2 : 26 478. This HC2 assignment has only been reported for isolates sourced in South Africa (from 2020 onwards), with the exception of a single isolate sourced in England in 2014 (European Nucleotide Archive biosample accession number SAMN03475542). This English case had reported travel history to Zimbabwe, a country which neighbours South Africa (M. A. Chattaway, UK Health Security Agency, personal communication, 23 February 2023). For cluster two isolates, at the HC2 level, isolates were assigned either HC2 : 256 191 (*n*=12) or HC2 : 295 929 (*n*=1). The HC2 : 256 191 assignment has only been reported for isolates sourced in South Africa (from 2020 onwards). For cluster three isolates, at the HC2 level, isolates were assigned either HC2 : 12 503 (*n*=13) or HC2 : 221 038 (*n*=1). The HC2 : 12 503 assignment has previously been reported for isolates sourced in South Africa (from 2010 onwards) and Malawi isolates (sourced from 2011 onwards). We further investigated genetic variation within clusters using a SNP analysis of isolates. Cluster one isolates showed 0–1 SNP differences, cluster two isolates showed 0–6 SNP differences, and cluster three isolates showed 0–3 SNP differences. The level of SNP variation within clusters showed concordance/congruence to the level of allele variation determined using cgMLST. These concordance of results have previously been reported when investigating clusters and outbreaks of enteric bacterial pathogens [18, 40–45].

*Salmonella* Typhi genotype 4.3.1 is commonly associated with AMR, and well-established in several African countries, including Tanzania, Kenya, Malawi, Zambia, Zimbabwe and South Africa [30, 39, 46]. Genotype 4.3.1 is subdivided into three major lineages (4.3.1.1, 4.3.1.2, and 4.3.1.3). All isolates associated with our Western Cape Province clusters showed a sublineage of 4.3.1.1, named genotype 4.3.1.1.EA1 (genotype 4.3.1 lineage I sublineage East Africa I). All isolates associated with our Western Cape Province clusters showed MDR, but thankfully, all isolates showed phenotypic susceptibility to ciprofloxacin, ceftriaxone and azithromycin, which are the agents currently recommended for the treatment of typhoid fever in South Africa [20].

Limitations of our study included the following. Although enteric fever is a notifiable medical condition in South Africa (such that all laboratory-confirmed cases must be reported to the Department of Health), reported cases significantly underrepresent the true number of cases. The likelihood that enteric fever cases are identified and diagnosed depends on many factors, including the severity of illness, whether health workers are aware of the disease, and whether blood culture capacity is available. WGS data is only available on isolates submitted by diagnostic laboratories to the reference laboratory at the CED, NICD; in a few instances isolates were not sent to the CED. Limitations of the epidemiologic investigations for these clusters included the inability to trace some of the close contacts (usually because of incorrect addresses or contact numbers), and that stool samples from food handlers working for the school feeding programme service provider were not collected.

In conclusion, we describe the use of WGS for surveillance and cluster detection of enteric fever in South Africa. Cluster identification allowed for targeted epidemiological investigations and timely, coordinated public health responses. Unfortunately, no confirmed source(s) of infection were identified for any of the clusters. Nonetheless, the public health response and outbreak investigations raised awareness of enteric fever among health officials, health workers and communities in the relevant districts and helped to inform control measures. Following the public health response, there was a marked decline in the number of cases of enteric fever diagnosed in these districts; it is likely that interventions implemented during the outbreak response, including health communication and enhanced water quality monitoring, contributed to this decline.

## Supplementary Data

Supplementary material 1
