# Supplementary material for: Enteric fever cluster identification in South Africa using genomic surveillance of Salmonella enterica serovar Typhi
Source: Microb Genom. 2023 Jun 20;9(6):mgen001044. doi: 10.1099/mgen.0.001044 (PMC10327498; doi:10.1099/mgen.0.001044)
Supplement: Supplementary material 1 [file mgen-9-1044-s001.pdf]

Enteric fever (typhoid and paratyphoid fever):

Recommendations for diagnosis, management and public health response

Developed by:
Centre for Enteric diseases
The National Institute for Communicable Diseases (NICD),
a division of the National Health Laboratory Service (NHLS)

Table of contents

Background on enteric fever **4**
1.1 Microbiology 4
1.2 Clinical features 4
1.3 Carriage 6
1.4 Transmission 7
1.5 Case definitions 7
1.6 Epidemiology 8
1.7 Antimicrobial resistance 8
2 Diagnosis **9**
2.1 Differential diagnosis 9
2.2 Sample collection for laboratory diagnosis 9
2.2.2 Stool culture 10
2.2.3 Bone marrow aspirate culture 10
2.2.4 Extra-intestinal complications 10
2.2.5 Urine cultures 11
1.1.6 Serology and other tests 11
3 Treatment and case management **11**
3.1 Antibiotic therapy 11
3.2 Uncomplicated enteric fever 12
3.3 Severe and complicated enteric fever 13
3.4 Treatment of carriage 14
3.5 Infection prevention and control 15
3.6 School and work restrictions 15
4 Public health response to a single case **15**
5 Public health response to a cluster or outbreak **17**
6 Prevention and control **17**
6.1 Safe water 17
6.2 Food safety 18
6.3 Sanitation 18
6.4 Vaccination 18
6.5 Travellers 19
7 Resources and additional information **20**
8 Appendix 1: Suspected/confirmed typhoid fever case investigation form **21**

Introduction

The aim of this guidance is to support healthcare and public health practitioners in the diagnosis, treatment and public health response to typhoid or paratyphoid infections.

Enteric fever (typhoid or paratyphoid fever) is a category 1 notifiable medical condition (NMC) in South Africa.
According to the National Health Act, 61 of 2003[1], category 1 NMCs require immediate reporting to health authorities, through the relevant electronic and/or paper based reporting platforms. Further information on the NMC notification process, notification forms and NMC case definitions can be found at https://www.nicd.ac.za/nmc-overview/overview/.

Refer to Health Act 61 of 2003. Available at: https://www.gov.za/documents/national -health-act (Accessed: 8 June 2022)

Background on enteric fever

1.1 Microbiology

Enteric fever includes typhoid fever (an infection caused by *Salmonella enterica* subspecies *enterica* serotype Typhi) and paratyphoid fever (infections caused by *Salmonella enterica* subspecies *enterica* serotypes Paratyphi A, B or rarely, C). Paratyphoid fever is clinically indistinguishable from typhoid fever and the diagnosis and clinical management is identical. For the purposes of this document, the term enteric fever encompasses infections caused by *S*. Typhi and *S*. Paratyphi A, B and C.

Whilst *S*. Paratyphi B causes enteric fever, there is a variant named *S*. Paratyphi B var. Java which is classified as a nontyphoidal *Salmonella* serotype and is associated with gastroenteritis. *S*. Paratyphi B var. Java can usually be distinguished from *S*. Paratyphi B through phenotypic testing.

1.2 Clinical features

Enteric fever is a systemic bacterial disease. The symptoms and clinical signs are nonspecific and overlap considerably with other causes of acute febrile illnesses. Disease manifests after an average incubation period of 9-21 days (range 3-60 days).

Acute enteric fever

Acute enteric fever is typically a systemic illness with fever as the cardinal characteristic. Fever is present in 97-100% of cases and is the first indication of illness, usually rising over a few days. Up to 44% of cases experience continuous fever, while 25% have a step-wise increase in fever, peaking in the second week of disease.

A range of other clinical features may be present, varying in frequency by age group (Table 1).

Table 1. Clinical features of enteric fever in children and adults

Clinical feature	Children	Adults
Gastrointestinal symptoms	Gastrointestinal symptoms vary, and are influenced by age:<br>• abdominal pain (70% in children 5-10 years; 20% in children < 5 years)<br>• nausea or vomiting (45% in children 5-10 years; 32% in children < 5 years)<br>• diarrhoea is >2.5 more common in infants than older children or adults	Gastrointestinal symptoms include<br>• Abdominal pain/cramps (54%)<br>• Nausea and vomiting (~50%)<br>• Diarrhoea (46%)<br>• constipation (24%)
Headache		63%
Hepatomegaly and splenomegaly	Hepatomegaly: 44-85%<br>Splenomegaly: 26-90%	Hepatomegaly: 30%<br>Splenomegaly: 40%
Cough	30-72%	41%
Relative bradycardia	11-30%	47%

Anaemia	Anaemia ~70% among children of all ages),	anaemia (30%) and
Leucocyte count	• Leucocytosis (47%) is more common than leucopenia (21%) in children 5-10 years of age. Leucopenia is more common than leucocytosis in children >10 years of age<br>• Eosinopenia: up to 70%	Leukopenia (36%) is more common than leucocytosis (13%)
Thrombocytopenia	<20%	53%
Hepatitis	26% children older than 5 years; 36% in children <5 years	28%
Seizures	More common in young children, particularly febrile seizures in 6 months to 5-year age-group	
Rose spots	Erythematous maculopapular lesions (rose spots): flat, faint pink spots 2-4cm in diameter which develop on the chest, abdomen and back. These occur in <7% of cases in low- and middle-income countries, but are reported more commonly in high-income countries (up to 19%) and may be difficult to detect in dark-skinned individuals.	

Severe and complicated illness

Untreated enteric fever can lead to severe, life-threatening complications which typically manifest 2-4 weeks after infection. Complications are estimated to occur in 27% of hospitalised children and 17% of hospitalised adults. Children with prolonged duration (≥10 days) of symptoms prior to hospitalisation are at three times higher risk of developing complications than those with symptoms for <10 days.

A wide range of complications has been described (Table 2). The complications with the highest risk for death include gastrointestinal bleeding, intestinal perforation, septic shock, and encephalopathy (often accompanied by shock). Of the other complications described, the most common include cholecystitis, hepatitis, pneumonia, acute kidney injury, and myocarditis.

Table 2. Complications of enteric fever

Complication
Intestinal
• Intestinal perforation (usually of ileum, occasionally of colon)
• Gastrointestinal bleeding
Extra-intestinal
• Central nervous system: encephalopathy (accompanied by shock) cerebral oedema, subdural empyema, cerebral abscess, meningitis, ventriculitis, transient parkinsonism, motor neuron disorders, ataxia, seizures, Guillain-Barré syndrome, psychosis
• Hepatobiliary system: cholecystitis, hepatitis, hepatic abscesses, splenic abscess, peritonitis, paralytic

	ileus, carcinoma of the gallbladder
•	Cardiovascular system: endocarditis, myocarditis, pericarditis, arteritis, congestive heart failure
•	Pulmonary system: pneumonia, empyema, bronchopleural fistula
•	Kidneys: acute kidney injury, nephritis
•	Bone and joint: osteomyelitis, septic arthritis
•	Genitourinary system: urinary tract infection, renal abscess, pelvic infections, testicular abscess, prostatitis, epididymitis
•	Soft tissue infections: psoas abscess, gluteal abscess, cutaneous vasculitis
•	Haematological: haemophagocytosis syndrome

Prior to the advent of antibiotics and in untreated cases, the case fatality ratio (CFR) due to enteric fever was reported as 10-20%. The CFR in endemic countries is now estimated at ~2.5% but increases to 4.5% in hospitalised patients. Antimicrobial resistance and the presence of complications increase the CFR. Intestinal perforation is reported to have the highest CFR (~15% on average, although sub-Saharan African countries tend to report higher CFRs of 20-30%). Children younger than 5 years of age have a four times higher risk of mortality than those older than 5 years.

Relapse of typhoid and paratyphoid fevers is uncommon, and usually occurs in patients who have not been treated or have been treated with chloramphenicol. Relapse manifests as a second episode of fever which occurs 1 – 3 weeks after recovery from the initial episode.

1.3 Carriage

About 10% of persons who have had an episode of enteric fever intermittently shed bacteria in faeces for several weeks after infection while up to 4% of those infected become chronic carriers. These individuals may excrete the organism for years if left untreated, with the potential to infect others, which has important implications for public health. Chronic faecal carriage is associated with gallstones, and is most common in women >40 years of age. Chronic urinary carriage is associated with schistosomiasis and nephrolithiasis. Most chronic carriers have no symptoms, and up to 25% give no history of acute enteric fever. Chronic carriage can occur with *S*. Typhi and *S*. Paratyphi A, but has not been described with *S*. Paratyphi B or C. Chronic carriers have an increased risk of carcinoma of the gallbladder.

Carriers are defined as follows:

Convalescent carrier: Evidence of shedding (positive stool culture) 1–12 months after finishing an appropriate course of antimicrobial treatment and the resolution of symptoms following a laboratory-confirmed episode of acute disease.

Definitive (chronic) carrier: Evidence of shedding (positive stool culture) at least 12 months after finishing an appropriate course of antimicrobial treatment and the resolution of symptoms following a laboratory-confirmed episode of acute disease OR two positive stool samples 12 months apart.

Presumptive carrier: Evidence of shedding (positive stool culture) of an unknown duration.

1.4 Transmission

Humans are the only known hosts of *S*. Typhi, *S*. Paratyphi A and *S*. Paratyphi C. There are reports of *S*. Paratyphi B associated with cattle, but this is uncommon. Bacteria are shed in the faeces of an infected person and transmitted from person to person via ingestion of food or water contaminated by faeces. Important sources of contaminated water include: surface water (e.g. rivers, dams, wells) contaminated with faeces or sewage; inadequately treated piped water; and contamination of treated drinking water (e.g. contamination of the bucket-and-cup household water storage system by an infected person). Large outbreaks of enteric fever are often associated with contamination of a drinking water source. The organism can survive for several days in fresh water (e.g. ground water, pond-water) and seawater and for prolonged periods (up to several months) in contaminated foods. Foodborne outbreaks have been associated with a range of food items, including eggs, oysters (fresh and frozen), ice-cream and iced-drinks, raw fruits and vegetables, fish and various meats. Contamination of food can occur through infected food handlers (any persons whose occupation involves the handling of food) or persons who prepare or handle food in a household setting, and irrigation of gardens/crops with sewage-contaminated water or fertilisers. Persons with occupations as food handlers (preparing or serving unwrapped food to be served raw or not subjected to further heating) or care-givers (particularly those caring for young children, the elderly, or other particularly vulnerable people whose activities increase the risk of transferring infection via the faeco-oral route), represent specific high-risk groups due to their potential to transmit infection widely. All children aged 5 years or younger attending pre-school, nursery or child-minding groups and individuals with unsatisfactory toilet or handwashing facilities are also at higher risk of transmitting gastrointestinal pathogens.

1.5 Case definitions

Because enteric fever presents as a nonspecific febrile illness, heightened clinical awareness and appropriate laboratory tests are critical to identify cases.

Enteric fever case definitions per the World Health Organization surveillance standards, are summarised in Table 3.

Table 3: Enteric fever case definitions

**Suspected case**	A person presenting with fever for at least three out of seven consecutive days <u>and</u> symptoms compatible with enteric fever
**Confirmed case**	Isolation of *Salmonella* Typhi or *Salmonella* Paratyphi A,B or C from a clinical sample
**Probable case (relevant only in outbreaks)**	A clinically compatible case that is epidemiologically linked to a confirmed case

Symptoms and signs suggestive of enteric fever include:

- Headache
- Gastrointestinal symptoms (abdominal pain, nausea and vomiting, diarrhoea or constipation)
- Relative bradycardia
- ‘Rose spots’ (erythematous maculopapular lesions)
- Splenomegaly and/or hepatomegaly
- Dry cough

- Leucopenia (low white cell count) or leucocytosis (high white cell count)
- Anaemia
- Thrombocytopenia
- Hepatitis

1.6 Epidemiology

Global

While the incidence of enteric fever has decreased worldwide over the last few decades, an estimated 11-20 million enteric fever cases with ~130 000 – 160 000 deaths occur each year. The incidence is highest in developing countries within Asia and parts of Africa.

Enteric fever remains endemic in many low- and middle-income countries, where asymptomatic carriers act as reservoirs and unsafe water and unimproved sanitation facilities and poor hygiene drive ongoing transmission. Children are disproportionately affected; the highest incidence rates typically occur among children 5-15 years of age, but recent studies from Africa and Asia have highlighted the substantial burden of disease among children less than 5 years of age and infants.

S. Typhi is the most common cause of enteric fever worldwide, accounting for ~76% of global cases in 2017. An increasing burden of *S.* Paratyphi A has been described across some countries in Asia (including Nepal, Cambodia and China).

South Africa

Enteric fever is endemic in South Africa and sporadic cases are reported from most provinces every year. Following the enteric fever outbreaks in 2005-2006, the number of culture-confirmed cases has remained stable with less than 150 cases per year. Most cases are locally acquired because of ongoing low-level transmission in affected communities, but imported travel-related cases are also identified. Although most cases are sporadic, small clusters and, infrequently, larger outbreaks do occur. Although persons of all ages are at risk for disease, children aged 5 to 14 years are usually most affected. There are usually more cases in the summer months. Paratyphoid fever remains uncommon in South Africa, with infrequent travel-related cases reported.

1.7 Antimicrobial resistance

Global

The emergence of multidrug resistant (MDR) *S*. Typhi, defined as resistance to first line antibiotics (chloramphenicol, ampicillin and cotrimoxazole), was first reported in Asia in the 1980's and subsequently became widespread. With the spread of MDR strains, ciprofloxacin was adopted as the treatment of choice. However, resistance to ciprofloxacin rapidly appeared (in the early 1990s), with the subsequent emergence of *S*. Typhi strains which were MDR with fluoroquinolone resistance. Third-generation cephalosporins, macrolides and carbapenems then increasingly became the agents of choice for treating enteric fever. Extensively drug resistant (XDR) *S*. Typhi (defined as resistance to the first-line antibiotics, a fluoroquinolone and a third-generation cephalosporin) was first reported in Pakistan in 2016, when it caused a large outbreak. XDR *S*. Typhi is geographically confined to Pakistan at present, but travel-related cases have been reported from several countries. Azithromycin is the only remaining oral drug to treat XDR typhoid fever, and the emergence of azithromycin-resistant *S*. Typhi in Bangladesh, Pakistan, Nepal and India is a serious concern.

There is wide variation in the rates of antimicrobial resistance across regions, and even across countries or areas within regions. MDR is uncommon in *S*. Paratyphi A strains.

<u>South Africa</u>

In South Africa, MDR typhoid fever was first described in 1987. Decreasing susceptibility to ciprofloxacin has been of particular concern over the last decade, but since 2015 the percentage of isolates submitted to the NICD for laboratory surveillance not susceptible to ciprofloxacin has remained stable (~16% on average). In 2020, 12/69 (17%) of tested *S*. Typhi isolates submitted to the NICD for laboratory surveillance were not susceptible to ciprofloxacin. All endemic isolates remain susceptible to azithromycin.

2 Diagnosis

2.1 Differential diagnosis

Enteric fever may be clinically indistinguishable from other causes of an enteric-fever like syndrome, including *Yersinia enterocolitica*, *Yersinia pseudotuberculosis, Campylobacter fetus* or other nontyphoidal *Salmonella* infections. Therefore, laboratory testing is advisable in all patients presenting with clinically compatible characteristics of enteric fever (see Section 1.2 above).

Other causes of febrile illness, and possibly concurrent infections, in returning travellers should also be considered e.g. malaria, dengue, hepatitis, etc.

2.2 Sample collection for laboratory diagnosis

The definitive diagnosis of enteric fever requires the isolation of *S.* Typhi or *S.* Paratyphi A, B or C from blood, bone marrow, faeces, urine or a sterile site. Bacterial isolation from bone marrow is historically the gold standard for the definitive diagnosis of enteric fever, with a suggested sensitivity of ~90% after 4 days of culture. However, bone marrow biopsies are invasive specialised procedures; therefore, blood culture is the recommended diagnostic test for enteric fever.

2.2.1 Blood culture

Blood is the preferred clinical sample for the diagnosis of enteric fever. While blood culture is the most common method for laboratory confirmation, it has several limitations including relatively poor sensitivity, particularly when only one sample is collected and there is extensive use of antimicrobials prior to health centre or hospital presentation.

The diagnostic sensitivity of blood culture is influenced by several factors, including the blood sample volume, the timing of the test in relation to the duration of illness, laboratory culture incubation conditions and prior treatment with antibiotics. Bacterial load in acute enteric is low, an average of < 1 cfu/mL of blood, and is maximal during the first week of illness. Recent systematic reviews have reported estimated blood culture sensitivities of 59% - 66% when compared to bone marrow results. Lower blood culture sensitivity was reported for lower blood sample volumes (51% for 2 mL samples compared to 65% for 10 mL samples), among patients with prior antibiotic use (34% lower) and among patients with symptoms for >1 week (31% lower).

A blood culture set consists of all bottles procured from a single venepuncture or during one catheter draw. Sensitivity is improved by collecting at least two sets of blood cultures (1 aerobic and 1 anaerobic bottle per set).

Recommendations for blood culture in the diagnosis of enteric fever include:

- Collecting the blood sample for culture as early as possible in the course of the disease
- Collecting the blood sample for culture prior to administration of antimicrobials whenever possible (but do not delay necessary care for critically ill patients)
- Ensure that the volume of blood is optimal for inoculation in broth culture bottles. Commercially prepared blood culture bottles include instructions to determine the appropriate volume of blood to be inoculated into each bottle, determined by age or weight.
- The timing of blood culture orders should be dictated by patient acuity. In urgent situations, two or more blood culture sets can be obtained sequentially over a short time interval (minutes), after which empiric therapy can be initiated. In less urgent situations, obtaining blood culture sets may be spaced over several hours or more.
- In cases where the patient is receiving antimicrobial therapy, specialised media with antibiotic neutralisation capabilities should be used.
- Blood culture bottles should be transported to the microbiology laboratory immediately. Bottles should be kept at room temperature during transport and should never be refrigerated after inoculation. If transportation to the laboratory is likely to be delayed, contact the laboratory for guidance on interim storage of inoculated bottles.

2.2.2 Stool culture

The isolation of *S.* Typhi or *S*. Paratyphi A, B or C from stool is suggestive of acute enteric fever when associated with a clinically compatible illness; however, stool culture is not recommended for the routine diagnosis of acute enteric fever and does not replace blood culture. Stool culture is less sensitive than blood culture and is usually only positive from the second week of illness. Stool culture is indicated for the follow up of cases after completion of treatment to monitor shedding and document clearance, or to screen for carriage in contacts.

Where indicated, stool samples may be submitted for culture as per standard procedures. The sample should ideally be processed immediately; if processing is likely to be delayed by more than one hour then the sample should be submitted in a transport medium (e.g. Cary-Blair or Amies).

A stool sample is always preferable to a rectal swab sample, but if the patient is unable to produce a stool sample then a rectal swab sample can be collected.

2.2.3 Bone marrow aspirate culture

Although bone marrow culture is considered the most sensitive of the clinical samples for isolation of enteric fever, bone marrow aspirations are invasive, specialised procedures and bone marrow culture is therefore not routinely recommended to diagnose enteric fever. A bone marrow aspirate may be indicated in patients who have been previously treated, who have a long history of illness and for whom there have been negative sets of blood cultures with the recommended volumes of blood. Furthermore, bone marrow aspirates collected for other indications (e.g. investigation of cytopenias) may be subjected to culture.

2.2.4 Extra-intestinal complications

In patients who present with extra-intestinal complications (e.g. endocarditis, pneumonia, meningitis, arthritis or focal abscesses) an appropriate sample from the site of focal infection may be submitted for culture. For

example, these may include: sputum, CSF, synovial fluid or pus. Blood cultures should still be obtained for these patients, and laboratory results should be interpreted in line with the patient's clinical presentation/history.

2.2.5 Urine cultures

S. Typhi and *S*. Paratyphi A may be excreted in urine during acute enteric fever, and cases of chronic urinary carriage do occur. Therefore, although not routinely suggested for laboratory diagnosis, the isolation of *S.* Typhi or *S*. Paratyphi A from urine is indicative of acute enteric fever or chronic carriage depending on the clinical presentation of the patient.

1.1.6 Serology and other tests

In line with current WHO guidelines, serological tests (such as Widal test) are not recommended for the diagnosis of enteric fever due to poor sensitivity and inadequate specificity.

3 Treatment and case management

Early diagnosis and prompt administration of appropriate antimicrobial therapy shortens the illness and reduces mortality from the complications of enteric fever. The choice of antimicrobial therapy depends on the susceptibility of enteric fever strains in the area of residence or travel. Obtaining a thorough patient history including recent travel assists in the choice of empiric treatment. Susceptibility results from culture are critical in guiding treatment.

Patients with uncomplicated enteric fever can be managed at home with oral antibiotics, reliable care and close medical follow-up for complications or failure to respond to therapy. Patients with persistent vomiting, severe diarrhoea, or the presence of complications (intestinal or extra-intestinal) require hospitalisation.

Supportive measures, such as oral or intravenous hydration, the use of antipyretics, appropriate nutrition etc. also play an important role where indicated.

3.1 Antibiotic therapy

Antibiotic therapy should be started if there is a clinical diagnosis of suspected enteric fever. Blood cultures must be collected before starting antibiotics where possible; however, this should not delay necessary care for critically ill patients. The blood sample for culture should be collected as early as possible in the course of the disease. Do not wait for laboratory results before starting antibiotic therapy.

The choice of antibiotic therapy depends on the severity of illness as well as the antimicrobial susceptibility profile of the infecting organism.

The WHO Expert Committee on Selection and Use of Essential Medicines published updated recommendations for antibiotic therapy for enteric fever in 2019. The committee endorsed ciprofloxacin, ceftriaxone and azithromycin as first-choice treatments for enteric fever in the WHO list of essential medicines (EML) as well as the list of essential medicines for children (EMLc).

Ciprofloxacin is recommended as first-choice in settings with low prevalence of fluoroquinolone resistance, while ceftriaxone and azithromycin are recommended first-choice treatments in settings where there is a high prevalence of fluoroquinolone resistance.

Treatment options for extensively drug-resistant enteric fever are limited to azithromycin (for uncomplicated disease) and meropenem (for severe disease).

3.2 Uncomplicated enteric fever

Patients with uncomplicated enteric fever can be managed at home with oral antibiotics, reliable care and close medical follow-up for complications or failure to respond to therapy.

Ciprofloxacin is the empiric treatment of choice for uncomplicated enteric fever in South Africa. Ciprofloxacin offers several advantages, including rapid clearance of fever and symptoms, low rates of post-treatment carriage, oral administration, and availability at most healthcare facilities.

When antimicrobial susceptibility results are available, the treatment can be tailored accordingly (Table 1).

Table 1: Recommended antimicrobial treatment for uncomplicated enteric fever according to antibiotic susceptibility profile*

		Paediatrics		Adults	
**Susceptibility**	**Antibiotic**	**Dose**	**Days**	**Dose**	**Days**
Susceptible to ciprofloxacin	Ciprofloxacin	20 mg/kg/day po in two divided doses (i.e. 12 hourly)	7	20 mg/kg/day po in two divided doses (i.e. 12 hourly);<br>500-750 mg po 12 hourly	7
Not susceptible to ciprofloxacin†	<u>Neonates</u><br>Cefotaxime	40-80 mg/kg/day IV in 2 divided doses (i.e. 12 hourly)	10-14	1-2 g IV 12 hourly	10-14
	<u>Children 1 month and over</u><br>Ceftriaxone	50-75 mg/kg/day IV in two divided doses (i.e. 12 hourly)	10-14		
	OR				
	Azithromycin	20 mg/kg po daily	7	20 mg/kg po daily (maximum 1000 mg/day)	7

Extensively drug resistant‡	Azithromycin	20 mg/kg po daily	7	20 mg/kg po daily (maximum 1000 mg/day)	7

*Susceptibility as determined using up to date CLSI/EUCAST breakpoints for *Salmonella* spp
†Not susceptible to ciprofloxacin, but susceptible to ceftriaxone
‡Extensively drug resistant: resistant to chloramphenicol, amoxicillin, cotrimoxazole, ciprofloxacin and ceftriaxone

3.3 Severe and complicated enteric fever

Severe enteric fever, patients unable to tolerate oral treatment due to vomiting and/or severe diarrhoea, and/or patients with intestinal/extra-intestinal complications typically require hospitalisation and parenteral antimicrobial treatment.

Intravenous ceftriaxone/cefotaxime or ciprofloxacin (intravenous or oral) is recommended as empiric treatment for severe and complicated enteric fever in South Africa. When antimicrobial susceptibility results are available, the treatment can be tailored accordingly (Table 2).

Once patients have clinically improved, treatment can be completed with appropriate oral antibiotics (e.g. oral ciprofloxacin or azithromycin, depending on the antimicrobial susceptibility profile).

Table 2: Recommended antimicrobial treatment for severe or complicated enteric fever according to antibiotic susceptibility profile*

		Paediatrics		Adults	
**Susceptibility**	**Antibiotic**	**Dose**	**Duration (days)**	**Dose**	**Duration (Days)**
Susceptible to ciprofloxacin	Ciprofloxacin†	10 mg/kg/dose (up to a maximum dose of 400 mg) IV 8 hourly<br>OR<br>20 mg/kg/day po in two divided doses (i.e. 12 hourly)	10-14	400 mg IV 8 hourly<br>OR<br>20 mg/kg/day po in two divided doses (i.e. 12 hourly); 500-750 mg po 12 hourly	10-14

Not susceptible to ciprofloxacin‡	Neonates<br><br>Cefotaxime<br><br>Children 1 month and over<br><br>Ceftriaxone	 <br>40-80 mg/kg/day IV in 2 divided doses (i.e. 12 hourly)<br><br>50-75 mg/kg/day IV in two divided doses (i.e. 12 hourly)	 <br>10-14<br><br>10-14	1-2 g IV 12 hourly	10-14
Extensively drug resistant§	Meropenem	For children 3 months and older: 20 mg/kg (up to maximum dose of 1g) IV 8 hourly	10-14	1 g IV 8 hourly	10-14

* Susceptibility as determined using up to date CLSI/EUCAST breakpoints for *Salmonella* spp
†Oral or intravenous ciprofloxacin may be used for severe disease
‡ Not susceptible to ciprofloxacin, but susceptible to ceftriaxone
§Extensively drug resistant: resistant to chloramphenicol, amoxicillin, cotrimoxazole, ciprofloxacin and ceftriaxone

Intestinal bleeding, perforations or ulcerations are life-threatening and may require immediate fluid resuscitation, surgical interventions and broad-spectrum antimicrobial coverage for polymicrobial peritonitis.

Data are limited, but concurrent treatment with high-dose dexamethasone (initial dose 3mg/kg followed by eight doses of 1mg/kg every 6 hours for 48 hours) should be considered for patients with severe enteric fever with delirium, obtundation, coma or shock. Administering dexamethasone has been shown to reduce fatalities among such patients; however, patients must be monitored closely because dexamethasone may mask abdominal complications.

In patients with severe enteric fever with intestinal perforation and peritonitis, surgical intervention is recommended.

3.4 Treatment of carriage

Patients with documented convalescent or chronic carriage should be treated for a prolonged duration with an appropriate oral antimicrobial.

If the infecting organism is susceptible to ciprofloxacin, the recommended treatment is ciprofloxacin (20 mg/kg/day po in two divided doses) for 28 days.

At present, there are no data as to which treatment is optimal for treating carriers with *S*. Typhi strains that are not susceptible to ciprofloxacin, and expert advice should be sought in such cases.

Investigation and management of anatomic abnormalities and concurrent infections also plays an important role in the management of chronic carriers. Antibiotics are less effective in patients with gallbladder, biliary or kidney stones. In these patients, surgery (e.g. cholecystectomy) combined with antibiotic therapy may be indicated. Concurrent schistosomiasis plays a role in the development of urinary carriage, and should be treated with praziquantel to eradicate *Schistosoma* spp. prior to initiating antibiotic therapy.

3.5 Infection prevention and control

Hospitalised patients should be cared for using standard precautions. Contact precautions should be used for diapered or incontinent persons for the duration of illness or to control institutional outbreaks.

It is important to educate the case and care givers with regards to the preventive steps to reduce the risk of transmission, including food safety and hand hygiene.

3.6 School and work restrictions

Cases that do not require hospital admission for treatment, should be restricted from school/work until 48 hours after symptoms subside, in order to minimise the risks for transmission. Based on risk assessment, redeployment or exclusion from high risk activities may be advised for groups at high risk of transmission pending treatment and clearance.

4 Public health response to a single case

All cases of enteric fever infection pose a public health risk in terms of spreading infection and potentially causing an outbreak. The following steps should be followed by healthcare workers following the identification of a suspected or confirmed case.

1. **Notify the Department of Health:** Enteric fever is a category 1 notifiable medical condition(NMC). Therefore, all healthcare professionals are required by law to notify any suspected or confirmed case of enteric fever to their local authority and Department of Health using the NMC system - paper-based form/electronic platform.
2. **Confirm the diagnosis:** Verify patient details and review laboratory results with reference to Section 2. Request a blood culture if a laboratory diagnosis has not yet been obtained or if a non-confirmatory test has been done (e.g. serology). For diagnostic support, contact the relevant microbiology laboratory.
3. **Review case management and treatment:** If enteric is suspected, commence treatment immediately (preferably after collection of blood cultures). Ensure the case is receiving the appropriate treatment for enteric fever and any concomitant infections**.** Exclude other causes of febrile illness, particularly malaria.
4. **Interview the case and complete a Case Investigation Form (CIF)** (Appendix 1) to ascertain risk factors for exposure and possible source(s) of infection. Send the CIF to the Provincial and District Communicable Disease Control and Surveillance focal person. It is especially important to obtain the following information:
    a. **Occupation.** Persons with occupations as food handlers or care-givers (particularly those caring for children or the elderly), represent specific high-risk groups due to their potential to transmit infection widely
    b. **Place of work or crèche/school if the case is a child**
    c. **Source of infection.** Investigate the source of infection, perform additional environmental investigations where indicated and intervene where a source can be identified.

5. **Educate the case and care-givers:** Conduct health promotion to educate the case/care-givers about enteric fever infection and transmission. Emphasise the importance of good hygiene practices, in particular hand

washing (especially before eating and preparing food, and after going to the toilet), safe sanitation and water use, and food safety.

6. **Case follow-up:** Follow-up the case after completion of antibiotic treatment with three stool samples as outlined below to confirm that s/he is not a carrier.

Figure 1: Follow-up of laboratory-confirmed enteric fever case following treatment

Collect three clearance stool samples for *S.* Typhi culture. Rectal swabs may be collected if stool cannot be obtained. The first sample should be obtained one week after completion of antibiotics. Subsequent samples should be collected 48 hours apart. If case originally had a positive urine culture, a history of urinary tract infection and/or a history of schistosomiasis (bilharzia), collect urine samples for culture in addition to stool samples.

All three cultures negative: If three consecutive cultures are all negative as per above criteria → release the case from surveillance

Any of the three samples culture-positive:

1. Consider retreatment (second course of antibiotics see section 3.2 Table 1)
2. Collect another three clearance stool samples (first sample one week after completing antibiotics; subsequent samples collected 48 hours apart).

All three cultures negative: If three consecutive cultures are all negative → release the case from surveillance

Any of the three samples culture-positive

Convalescent carrier:

1. Consider 4-6 weeks of treatment with appropriate antimicrobial (see section 3.4)
2. Repeat stool samples monthly.

A negative monthly sample

Collect two further samples 48 hours apart

Positive monthly samples

1. Continue monthly stool sample collection
2. Consider investigation for possible causes of prolonged carriage (see section 1.2)

All three samples negative

→ release the case from surveillance

Positive monthly samples after 12 months of repeat sampling: Chronic carrier
Refer for specialist opinion and management.

7. **Contact management:** Identify all contacts at risk of infection, which may include: household members, care givers of the case, and people who may have eaten the implicated food/water/beverages. The following response should be completed for all contacts at risk of infection:
   a. Collect a stool/rectal swab sample for *S.* Typhi culture.
   b. Interview all contacts, completing the line-list at the end of the case questionnaire (Appendix 1)
   c. Educate all contacts on enteric fever infection, transmission, prevention, and recognising symptoms and seeking medical care if these occur.
   d. If a contact's stool/rectal swab is culture positive, refer the person for antibiotic treatment and complete the response steps 1-7 for that person. Identification of *S.* Typhi from stool is suggestive of enteric fever when associated with a clinically compatible illness. *S.* Typhi in the absence of clinical illness may be suggestive of *S.* Typhi carriage.

5 Public health response to a cluster or outbreak

An outbreak of enteric fever is defined as two or more epidemiologically-linked suspected or confirmed cases. All outbreaks must be immediately notified to health authorities for investigation and response. These activities should be coordinated by the local level Department of Health offices in collaboration with stakeholders, which may include:

- District, Provincial and National Department of Health: Communicable Disease Control, Environmental Health, Epidemiology and Surveillance, Disaster Management, Infection Control, etc.
- NHLS diagnostic laboratories, NICD - Centre for Enteric Diseases (CED), private laboratories.
- Inter-sectoral stakeholders when required, e.g.: Department of Water Affairs, Department of Agriculture, Department of Education, etc.

6 Prevention and control

The prevention of enteric fever is primarily based on ensuring access to safe water, food safety and proper sanitation infrastructures. Health promotion and education is paramount to raising public awareness about these practices and inducing behaviour change within a community. This intervention should especially be implemented for each and every known case and his/her contacts. Other public health measures include accurate and timely diagnosis, appropriate antimicrobial treatment and identification and treatment of chronic carriers. Vaccination against typhoid fever is available in South Africa; however, this is only indicated for laboratory staff that work regularly with the pathogen and may also be considered for travellers for highly endemic settings. The limitations of these vaccines should be noted and should not detract against the primary measures of preventing infections.

6.1 Safe water

Both sporadic cases and outbreaks of enteric fever have been associated with poor quality water for drinking and other domestic activities (cooking, washing, etc.). An overall reduction in disease burden may be achieved through ensuring access to safe water throughout the community. Likewise, provision of safe water may play a

role in controlling outbreaks (when suspected as the source) as well as preventing additional settings within the household setting. Response measures may include:

- **In areas with access to municipal water systems:** enhanced monitoring of water throughout the supply systems (from point of treatment to consumer outlets), and ensuring appropriate water treatment (e.g. adequate chlorine concentrations to disinfect the water).
- **In areas without access to treated water:**
    - Monitoring of drinking water sources, and treating of these if practical (e.g. in the case of JoJo tanks, wells).
    - Provision of alternative water sources (e.g. supply safe/treated water using water-tankers, JoJo tanks)
    - Distribute resources and conduct health promotion activates for point-of-use disinfection water within households. Disinfection methods may include boiling and/or chemical disinfectants (chlorine bleach, tablets, etc.). Safe storage and use of water also plays an important role in preventing secondary spread in households (for example: use plastic narrow-mouthed containers with covers to avoid recontamination after treatment).

6.2 Food safety

Contaminated food plays and important role in the transmission of enteric fever. Following identification of a case, food safety should be promoted with households to prevent transmission to close contacts. This should include:

- Wash hands with soap and clean water before preparing and eating food, and after going to the toilet.
- Wash all surfaces and equipment used for food preparation with soap and clean water.
- Cook food thoroughly, avoid raw (uncooked) food (especially shellfish and meats). Eat only cooked and still hot food or reheat it.
- Use only safe water for preparing food, beverages and ice (or treat water before using).
- Wash and peel all fruit and vegetables before eating (especially when eaten raw)
- Do not use fertilisers that contain human-waste (excreta or faeces) on vegetable gardens or crops.

Based on risk assessment, redeployment or exclusion from high risk activities may be advised for acute cases and proven carriers who handle, process and/or serve food pending treatment and clearance.

In outbreak situations, food safety behaviours should be reinforced at a community level. Food safety inspections at restaurants and street vendors, and ensuring compliance with regulations, will also play an important role in preventing infections.

6.3 Sanitation

Provision of proper sanitation infrastructures will also reduce the burden of enteric fever, as well as other enteric diseases, within a community. Ensure appropriate systems for human-waste disposal and sewage treatment for all community members, monitor these systems continually, and maintain proper functioning at all times. In areas without municipal sewage systems, toilets (e.g. pit-latrines) should be built, regularly serviced and maintained to ensure safe functioning. Restrict access of the general public to sanitation infrastructure to prevent human-excreta from being used as fertilisers. Rapid provision of safe sanitation infrastructures (e.g. building pit-latrines) or investigating, and fixing faults in existing sanitation systems, may also play a role in controlling outbreaks.

6.4 Vaccination

Globally, three types of typhoid vaccines are currently licensed for use: typhoid conjugate vaccine, unconjugated Vi polysaccharide vaccine and live attenuated Ty21a vaccine. The WHO recommends their programmatic use for the control of typhoid fever when implemented together with interventions targeting

water, sanitation and hygiene, health education and other public health measures. These typhoid vaccines do not confer protection against *S*. Paratyphi A, but several bivalent *S*. Typhi and *S*. Paratyphi A vaccines are currently in development.

Routine typhoid vaccination is currently not recommended in South Africa. Vaccination is indicated for laboratory staff that work regularly with *S.* Typhi. It may also be considered for travellers to highly endemic countries.

The Vi purified polysaccharide antigen vaccine is currently the only typhoid vaccine registered for use in South Africa. It is licenced for use in persons aged 2 years and older. This vaccine is administered as single dose (intramuscular or deep subcutaneous) and becomes effective 2-3 weeks after injection. In pre-licensure trails, vaccine efficacy was 64% [95% CI:36-79] over 21 months of follow-up. Protection lasts at least 3 years. Data on safety and immunogenicity in pregnant women are currently lacking, however there are no theoretical safety concerns for the unconjugated Vi polysaccharide vaccine. This vaccine does not protect against *S.* Paratyphi infection. and shows reduced immunogenicity in children younger than 2 years of age.

WHO recommends vaccination in response to confirmed outbreaks of typhoid fever; however, data on the use of typhoid vaccines for outbreak control are very limited. Factors such as vaccine availability, logistics, cost and characteristics of the outbreak should be taken into consideration. Such campaigns should not detract from the primary control interventions (i.e. safe water, food safety and proper sanitation)

6.5 Travellers

Travellers should be advised to:

- Drink only water that is bottled or bring it to a rolling boil for at least 1 minute. Bottled carbonated water is generally safer than un-carbonated water.
- Avoid ice and food products (e.g. ice cream) that are potentially made with contaminated water.
- Eat foods that have been thoroughly cooked and that are hot and steaming. Avoid raw vegetables and fruits that cannot be peeled.
- Peel the fruit and vegetables yourself after washing your hands with soap. Do not eat the peelings.
- Avoid foods and beverages from street vendors and informal sellers.

Travellers to highly endemic areas (including south-central Asia, south-east Asia and parts of Africa), or areas with ongoing enteric fever outbreaks, may consider vaccination. These may be obtained from most travel clinics and should be given at least 2 weeks before departure. Given the limitations of vaccination, it is important to emphasise scrupulous personal, food and water hygiene at all times during travel.

7 Resources and additional information

Further questions from health professionals can be addressed to:

- The NICD Hotline +27 800212552 ***strictly for use by health professionals only***

Additional information on enteric fever is available from the following references:

- Centers for Disease Control and Prevention (CDC). Typhoid fever and paratyphoid fever. Information for Healthcare professionals Available online: https://www.cdc.gov/typhoid-fever/health-professional.html Last accessed 7 June 2022

- World Health Organization (WHO). Typhoid vaccines: WHO position paper. Weekly epidemiological record, 2018:93(13);153-172. Available online: https://www.who.int/publications/i/item/typhoid-vaccines-who-position-paper-march-2018 Last accessed 7 June 2022

- Public Health England. Interim-Public Health Operational Guidelines for Typhoid and Paratyphoid (Enteric Fever). A joint guideline from Public Health England and the Chartered Institute of Environmental Health. 2017. Available online: https://www.gov.uk/government/publications/typhoid-and-paratyphoid-public-health-operational-guidelines Last accessed 7 June 2022

- World Health Organization. Typhoid. Available online: https://www.who.int/health-topics/typhoid#tab=tab Last accessed 7 June 2022

- Nabarro LE, McCann N, Herdman MT, Dugan C, Ladhani S, *et al*. British Infection Association guidelines for the diagnosis and management of enteric fever in England. Journal of Infection.2022;84:469-489. https://doi.org/10.1016/j.jinf.2022.01.014

8 Appendix 1: Suspected/confirmed typhoid fever case investigation form

Enteric Fever Case Investigation Form

Electronic copy of form available from https://www.nicd.ac.za/wp-content/uploads/2022/03/Enteric-Fever-CIF_7-Jan-2022_Final.pdf

INTERVIEWER DETAILS	
**1. Interviewer name:**	**2. Date of interview:** DD / MM / YYYY
**3. Interviewer phone no.:**	**4. Department:**

PATIENT DETAILS		
**5. First name & Surname:**		
**6. DOB/(Age):**	**7. Gender:** ☐ Male ☐ Female	
**8. Phone no.:**		
**9. Place of residence (in the last month before illness):**		
**Town/City:**	**District:**	**Province:**
**10. Occupation:**	**11. Place of Work:**	
**11.1 For children: Name of crèche/school attended:**		
**12. Works in a food handling trade?** ☐ Yes ☐ No<br>**13. Works in a child/elderly/health care-giving setting?** ☐ Yes ☐ No		

DISEASE PRESENTATION
**14. Date of onset?** DD / MM / YYYY
**15. Symptoms/Signs:** (tick all that apply)<br>☐ Fever ☐ Vomiting ☐ Abdominal Cramps ☐ Malaise/Fatigue<br>☐ Headache ☐ Constipation ☐ Myalgia ☐ Respiratory symptoms (e.g. cough)<br>☐ Nausea ☐ Diarrhoea ☐ Rose Spots (red macules/rash)<br>☐ Hepatomegaly (enlarged liver) ☐ Splenomegaly (enlarged spleen)<br>☐ Other, Specify: ____________________
**16. Complications** (tick all that apply): ☐ Intestinal bleed ☐ Intestinal perforation ☐ Renal failure<br>☐ Encephalopathy (altered mental state eg confusion, loss of consciousness, seizures)
**17. Outcome**: ☐ Recovered /Discharged ☐ Still ill /Still admitted ☐ Died Date of death: DD / MM / YYYY

CLINIC/HOSPITAL DETAILS	
**18. Name of the clinician:**	**19. Phone no.:**
**20. Facility name:**	**21. Date of 1st consultation:** DD / MM / YYYY
**22. Name of referring facility (if applicable):**	
**23. Admitted to hospital?** ☐ Yes ☐ No	

LABORATORY INVESTIGATIONS

24. Date of specimen collection: DD / MM / YYYY

25. Lab name: **26. Lab number:**

27. Test/s performed for enteric fever diagnosis: (tick all that apply) ☐ Blood Culture ☐ Stool Culture ☐ Other, specify:______________________

28. Follow up testing: (tick all tests performed)

☐ Stool Culture 1 Date collected:_______________ Result:: ☐ Pos ☐ Neg

☐ Stool Culture 2 Date collected:_______________ Result:: ☐ Pos ☐ Neg

☐ Stool Culture 3 Date collected:_______________ Result:: ☐ Pos ☐ Neg

☐ Additional/other follow-up tests, give details: ____

HIV STATUS and ART

29. What is the current HIV status? ☐ HIV-infected ☐ HIV-uninfected ☐ HIV-unexposed uninfected ☐ HIV-exposed uninfected ☐ Unknown

30: Currently on Anti-retroviral therapy (ART)? ☐ Yes ☐ No ☐ Unknown

If yes, date of initiation of ART : DD/MM/YY ☐ Unknown

31. Is the patient currently taking cotrimoxazole prophylaxis? ☐ Yes ☐ No ☐ Unknown

EXPOSURE QUESTIONS

32. Have you travelled outside of your home town/city within 1 month before your illness started? (include local and international travel) ☐ Yes ☐ No

If yes, list all places/countries visited: ____

date departed: DD / MM / YYYY **date returned:** DD / MM / YYYY

33. Have you had any visitors from outside your home town/city within 1 month before illness onset? (include local and international travel) ☐ Yes ☐ No

If yes, where did they come from: ____

34. Have any of your close contacts or household members presented with similar illness to yours in the 1 month before your illness started? ☐Yes ☐No

If yes, list names and contact details:

Name	Phone no.	Address

35. Have you eaten at any of the following places within 1 month before your illness started?

Type		Name/Address/Phone no.
Café / Restaurant	☐ Yes ☐ No	
Street vendor	☐ Yes ☐ No	
Fast food	☐ Yes ☐ No	
Other, specify:		

36. Gatherings: Have you attended any gatherings that included a meal (eg wedding, party, funeral) within 1 month before your illness started? ☐ Yes ☐ No

37. Housing type: ☐Formal housing ☐Dwelling outside house ☐Informal settlement ☐Traditional house ☐Hostel/Institution

38. Number of people living in the house: _______________

39.Main source of water in the household: ☐Tap inside ☐Tap outside ☐River/dam ☐Tank/Jojo ☐Borehole ☐Other (Specify)______________________________

40: Is your water source: ☐Private (only used by your family) ☐Communal (shared by multiple families known to you) ☐Public (shared by people known and unknown to you)

41: Do you treat your water before drinking? ☐Y ☐N

41.1: if yes, indicate how: ☐Boil ☐Chemical (Bleach/chlorine tablets) ☐Other (Specify)__________________

42. Main sanitation in the household: ☐ Flush toilet ☐Chemical toilet ☐Latrine ☐Bucket ☐None ☐Other (Specify)__________________________

43. Where is your toilet situated? ☐ Inside ☐Outside ☐Other (Specify)______________________ ________

44: Is your toilet: ☐Private (only used by your family) ☐Communal (shared by multiple families known to you) ☐Public (shared by people known and unknown to you)

45: Do you have the following in your dwelling?

Fridge ☐Yes ☐ No ☐ Unknown **Food Preparation area** ☐Yes ☐ No ☐ Unknown

Freezer ☐Yes ☐ No ☐ Unknown **Sink to wash hands** ☐Yes ☐ No ☐ Unknown

Soap for handwashing at the sink ☐Yes ☐ No ☐ Unknown

46. Do you store water in your home? ☐ Yes ☐ No

If yes, in what type of container is water stored? (tick all that apply)

☐ Plastic container ☐ Metal container ☐ Open container ☐ Closed container with lid

How is water removed from the container? (tick all that apply)

☐ With hands ☐ With a spoon/cup/jug ☐ With a tap ☐ Other, specify: _________________________

47. Who prepares most of the meals in your home? (name and relationship to case): ______________________________

Does he/she wash hands before preparing food? ☐Yes ☐No

Has he/she ever had a similar illness to yours? ☐Yes ☐No

48. Do you grow your own vegetables at home? ☐Yes ☐No

If yes, from where do you get the water for your vegetable garden? ____________________________________

What do you use to fertilise your vegetable garden?____

Additional notes / comments / actions taken:

ENVIRONMENTAL ASSESSMENT

45. List all environmental samples collected: (if applicable)

**Type of sample** (food/water/milk)	**Place / Address where collected**	**Lab no.**	**Result**

Name of lab(s) processing samples: ____

CONTACT TRACING

1.Identify contacts at risk of infection, including: household members, care-givers of the case, and people who may have eaten the implicated food or water/beverages.
2. Investigate all contacts as per guidelines. List all below:

**Name**	**Age (years)**	**Sex (M/F)**	**History of enteric fever (Y/N)**	**Occupation**	**Physical address**	**Stool sample collected (Y/N)**	**Lab number/result**	**Referred for treatment (Y/N)**

Enteric Fever Case Investigation Form

**INTERVIEWER DETAILS**	
**1. Interviewer name:**	**2. Date of interview:** DD / MM / YYYY
**3. Interviewer phone no.:**	**4. Department:**

**PATIENT DETAILS**		
**5. First name & Surname:**		
**6. DOB/(Age):**	**7. Gender:** ☐ Male ☐ Female	
**8. Phone no.:**		
**9. Place of residence (in the last month before illness):**		
**Town/City:**	**District:**	**Province:**
**10. Occupation:**	**11. Place of Work:**	
**11.1 For children: Name of crèche/school attended:**		
**12. Works in a food handling trade?** ☐ Yes ☐ No		
**13. Works in a child/elderly/health care-giving setting?** ☐ Yes ☐ No		

DISEASE PRESENTATION

14. Date of onset? DD / MM / YYYY

15. Symptoms/Signs: (tick all that apply)
☐ Fever ☐ Vomiting ☐ Abdominal Cramps ☐ Malaise/Fatigue
☐ Headache ☐ Constipation ☐ Myalgia ☐ Respiratory symptoms (e.g. cough)
☐ Nausea ☐ Diarrhoea ☐ Rose Spots (red macules/rash)
☐ Hepatomegaly (enlarged liver) ☐ Splenomegaly (enlarged spleen)
☐ Other, Specify: ____________________

16. Complications (tick all that apply): ☐ Intestinal bleed ☐ Intestinal perforation ☐ Renal failure
☐ Encephalopathy (altered mental state eg confusion, loss of consciousness, seizures)

17. Outcome: ☐ Recovered /Discharged ☐ Still ill /Still admitted ☐ Died Date of death: DD / MM / YYYY

**CLINIC/HOSPITAL DETAILS**	
**18. Name of the clinician:**	**19. Phone no.:**
**20. Facility name:**	**21. Date of 1st consultation:** DD / MM / YYYY
**22. Name of referring facility (if applicable):**	
**23. Admitted to hospital?** ☐ Yes ☐ No	

**LABORATORY INVESTIGATIONS**	
**24. Date of specimen collection:** DD / MM / YYYY	
**25. Lab name:**	**26. Lab number:**
**27. Test/s performed for enteric fever diagnosis: (tick all that apply)**	☐ Blood Culture ☐ Stool Culture ☐ Other, specify:____________

28. Follow up testing: (tick all tests performed)

☐ Stool Culture 1 Date collected:_______________ Result:: ☐ Pos ☐ Neg

☐ Stool Culture 2 Date collected:_______________ Result:: ☐ Pos ☐ Neg

☐ Stool Culture 3 Date collected:_______________ Result:: ☐ Pos ☐ Neg

☐ Additional/other follow-up tests, give details: ____

HIV STATUS and ART

29. What is the current HIV status? ☐ HIV-infected ☐ HIV-uninfected ☐ HIV-unexposed uninfected ☐ HIV-exposed uninfected ☐ Unknown

30: Currently on Anti-retroviral therapy (ART)? ☐ Yes ☐ No ☐ Unknown

If yes, date of initiation of ART : DD/MM/YY ☐ Unknown

31. Is the patient currently taking cotrimoxazole prophylaxis? ☐ Yes ☐ No ☐ Unknown

EXPOSURE QUESTIONS

32. Have you travelled outside of your home town/city within 1 month before your illness started? (include local and international travel) ☐ Yes ☐ No

If yes, list all places/countries visited: ____

date departed: DD / MM / YYYY **date returned:** DD / MM / YYYY

33. Have you had any visitors from outside your home town/city within 1 month before illness onset? (include local and international travel) ☐ Yes ☐ No

If yes, where did they come from: ____

34. Have any of your close contacts or household members presented with similar illness to yours in the 1 month before your illness started? ☐Yes ☐No

If yes, list names and contact details:

Name	Phone no.	Address

35. Have you eaten at any of the following places within 1 month before your illness started?

Type		Name/Address/Phone no.
Café / Restaurant	☐ Yes ☐ No	
Street vendor	☐ Yes ☐ No	
Fast food	☐ Yes ☐ No	
Other, specify:		

36. Gatherings: Have you attended any gatherings that included a meal (eg wedding, party, funeral) within 1 month before your illness started? ☐ Yes ☐ No

37. Housing type: ☐Formal housing ☐Dwelling outside house ☐Informal settlement ☐Traditional house ☐Hostel/Institution

38. Number of people living in the house: ______________

39.Main source of water in the household: ☐Tap inside ☐Tap outside ☐River/dam ☐Tank/Jojo ☐Borehole ☐Other (Specify)____________________________

40: Is your water source: ☐Private (only used by your family) ☐Communal (shared by multiple families known to you) ☐Public (shared by people known and unknown to you)

41: Do you treat your water before drinking? ☐Y ☐N

41.1: if yes, indicate how: ☐Boil ☐Chemical (Bleach/chlorine tablets) ☐Other (Specify)__________________

42. Main sanitation in the household: ☐ Flush toilet ☐Chemical toilet ☐Latrine ☐Bucket ☐None ☐Other (Specify)__________________________

43. Where is your toilet situated? ☐ Inside ☐Outside ☐Other (Specify)______________________ _______

44: Is your toilet: ☐Private (only used by your family) ☐Communal (shared by multiple families known to you) ☐Public (shared by people known and unknown to you)

45: Do you have the following in your dwelling?

Fridge ☐Yes ☐ No ☐ Unknown **Food Preparation area** ☐Yes ☐ No ☐ Unknown

Freezer ☐Yes ☐ No ☐ Unknown **Sink to wash hands** ☐Yes ☐ No ☐ Unknown

Soap for handwashing at the sink ☐Yes ☐ No ☐ Unknown

46. Do you store water in your home? ☐ Yes ☐ No

If yes, in what type of container is water stored? (tick all that apply)

☐ Plastic container ☐ Metal container ☐ Open container ☐ Closed container with lid

How is water removed from the container? (tick all that apply)

☐ With hands ☐ With a spoon/cup/jug ☐ With a tap ☐ Other, specify: _________________________

47. Who prepares most of the meals in your home? (name and relationship to case): ______________________________

Does he/she wash hands before preparing food? ☐Yes ☐No

Has he/she ever had a similar illness to yours? ☐Yes ☐No

48. Do you grow your own vegetables at home? ☐Yes ☐No

If yes, from where do you get the water for your vegetable garden? ______________________________________

What do you use to fertilise your vegetable garden?____

Additional notes / comments / actions taken:

ENVIRONMENTAL ASSESSMENT

45. List all environmental samples collected: (if applicable)

**Type of sample** (food/water/milk)	**Place / Address where collected**	**Lab no.**	**Result**

Name of lab(s) processing samples: ____

CONTACT TRACING

1.Identify contacts at risk of infection, including: household members, care-givers of the case, and people who may have eaten the implicated food or water/beverages.
2. Investigate all contacts as per guidelines. List all below:

**Name**	**Age (years)**	**Sex (M/F)**	**History of enteric fever (Y/N)**	**Occupation**	**Physical address**	**Stool sample collected (Y/N)**	**Lab number/result**	**Referred for treatment (Y/N)**

Metadata for *Salmonella* Typhi isolates, Western Cape Province, South Africa, 2020-2021

Laboratory number	Source	Sample type	Patient age (years)	Patient sex	Collection Year	Collection Month	Collection Day	Continent	Country	Province	Cluster	Bio Project ID	Sample ID	cgMLST	HC0 (indistinguishable)	HC2	HC5	HC10	HC20	HC50	HC100	HC200	HC400	HC900 (ceBG)	HC2000 (Super-lineage)	HC2600	HC2850 (subsp.)
YA00193733	Human	Stool	46	Male	2020	1	19	Africa	South Africa	Western Cape		PRJEB39546	SAMEA8598154	221037	221037	221037	202	202	202	202	202	202	202	202	202	2	2
YA00198597	Human	Stool	43	Male	2020	2	28	Africa	South Africa	Western Cape		PRJEB39546	SAMEA7176653	231670	231670	231670	202	202	202	202	202	202	202	202	202	2	2
YA00198605	Human	Stool	23	Male	2020	3	6	Africa	South Africa	Western Cape		PRJEB39546	SAMEA7176654	231671	231671	231671	231671	202	202	202	202	202	202	202	202	2	2
YA00202148	Human	Blood culture	13	Female	2020	3	5	Africa	South Africa	Western Cape		PRJEB39546	SAMEA7176359	231676	231676	231676	231676	30420	30420	992	202	202	202	202	202	2	2
YA00202151	Human	Blood culture	17	Male	2020	3	10	Africa	South Africa	Western Cape		PRJEB39546	SAMEA7176358	231678	231678	231676	231676	30420	30420	992	202	202	202	202	202	2	2
YA00202184	Human	Blood culture	11	Female	2020	3	25	Africa	South Africa	Western Cape		PRJEB39546	SAMEA7176362	231677	231677	231676	231676	30420	30420	992	202	202	202	202	202	2	2
YA00204772	Human	Blood culture	14	Female	2020	4	5	Africa	South Africa	Western Cape		PRJEB39546	SAMEA7176463	231673	231673	231673	155112	155112	154331	154331	202	202	202	202	202	2	2
YA00204930	Human	Stool	14	Female	2020	4	5	Africa	South Africa	Western Cape		PRJEB39546	SAMEA7176377	232476	231673	231673	155112	155112	154331	154331	202	202	202	202	202	2	2
YA00204934	Human	Blood culture	7	Female	2020	4	8	Africa	South Africa	Western Cape		PRJEB39546	SAMEA7176655	231671	231671	231671	231671	202	202	202	202	202	202	202	202	2	2
YA00214388	Human	Blood culture	11	Male	2020	8	3	Africa	South Africa	Western Cape		PRJEB39546	SAMEA8598383	246855	246855	12503	202	202	202	202	202	202	202	202	202	2	2
YA00214540	Human	Blood culture	22	Female	2020	8	9	Africa	South Africa	Western Cape		PRJEB39546	SAMEA8598369	246856	246856	246856	202	202	202	202	202	202	202	202	202	2	2
YA00216712	Human	Blood culture	28	Male	2020	8	26	Africa	South Africa	Western Cape		PRJEB39546	SAMEA10113648	248804	133397	133397	202	202	202	202	202	202	202	202	202	2	2
YA00217997	Human	Blood culture	20	Female	2020	9	16	Africa	South Africa	Western Cape		PRJEB39546	SAMEA8598112	254411	254411	37213	202	202	202	202	202	202	202	202	202	2	2
YA00220129	Human	Blood culture	41	Male	2020	10	7	Africa	South Africa	Western Cape	Cluster 1	PRJEB39546	SAMEA8598158	256057	249364	26478	26478	202	202	202	202	202	202	202	202	2	2
YA00221666	Human	Stool	14	Female	2020	10	13	Africa	South Africa	Western Cape	Cluster 1	PRJEB39546	SAMEA10113534	255634	249364	26478	26478	202	202	202	202	202	202	202	202	2	2
YA00221956	Human	Blood culture	13	Male	2020	10	13	Africa	South Africa	Western Cape	Cluster 1	PRJEB39546	SAMEA8598535	249364	249364	26478	26478	202	202	202	202	202	202	202	202	2	2
YA00221965	Human	Blood culture	45	Female	2020	10	20	Africa	South Africa	Western Cape	Cluster 1	PRJEB39546	SAMEA8598537	249365	249364	26478	26478	202	202	202	202	202	202	202	202	2	2
YA00223222	Human	Blood culture	11	Male	2020	11	5	Africa	South Africa	Western Cape	Cluster 1	PRJEB39546	SAMEA8598088	253382	249364	26478	26478	202	202	202	202	202	202	202	202	2	2
YA00223985	Human	Blood culture	18	Male	2020	11	11	Africa	South Africa	Western Cape	Cluster 1	PRJEB39546	SAMEA8598086	253383	253383	26478	26478	202	202	202	202	202	202	202	202	2	2
YA00223990	Human	Blood culture	11	Male	2020	10	20	Africa	South Africa	Western Cape	Cluster 1	PRJEB39546	SAMEA8598089	253389	249364	26478	26478	202	202	202	202	202	202	202	202	2	2
YA00224875	Human	Blood culture	12	Male	2020	11	16	Africa	South Africa	Western Cape	Cluster 3	PRJEB39546	SAMEA9966784	279844	279844	12503	202	202	202	202	202	202	202	202	202	2	2
YA00230158	Human	Blood culture	26	Male	2020	12	19	Africa	South Africa	Western Cape		PRJEB39546	SAMEA10113568	256188	256188	256188	30420	30420	30420	992	202	202	202	202	202	2	2
YA00230352	Human	Blood culture	4	Male	2020	12	10	Africa	South Africa	Western Cape	Cluster 1	PRJEB39546	SAMEA10113571	256187	249364	26478	26478	202	202	202	202	202	202	202	202	2	2
YA00231057	Human	Stool	10	Male	2020	8	28	Africa	South Africa	Western Cape	Cluster 2	PRJEB39546	SAMEA10113569	256191	256191	256191	202	202	202	202	202	202	202	202	202	2	2
YA00231359	Human	Blood culture	4	Female	2020	12	31	Africa	South Africa	Western Cape		PRJEB39546	SAMEA8598478	259111	259111	259111	259111	202	202	202	202	202	202	202	202	2	2
YA00237160	Human	Blood culture	13	Male	2021	1	27	Africa	South Africa	Western Cape	Cluster 1	PRJEB39546	SAMEA8598235	256635	249364	26478	26478	202	202	202	202	202	202	202	202	2	2
YA00239004	Human	Blood culture	39	Female	2020	2	14	Africa	South Africa	Western Cape		PRJEB39546	SAMEA9966846	280994	260211	260211	202	202	202	202	202	202	202	202	202	2	2
YA00242167	Human	Blood culture	36	Female	2021	3	5	Africa	South Africa	Western Cape		PRJEB39546	SAMEA8598854	260211	260211	260211	202	202	202	202	202	202	202	202	202	2	2
YA00244313	Human	Blood culture	7	Female	2021	3	1	Africa	South Africa	Western Cape	Cluster 3	PRJEB39546	SAMEA8598641	261323	261323	12503	202	202	202	202	202	202	202	202	202	2	2
YA00244322	Human	Blood culture	10	Male	2021	3	11	Africa	South Africa	Western Cape	Cluster 1	PRJEB39546	SAMEA8598665	261331	249364	26478	26478	202	202	202	202	202	202	202	202	2	2
YA00244716	Human	Blood culture	23	Female	2021	3	16	Africa	South Africa	Western Cape		PRJEB39546	SAMEA8598663	221026	221026	208099	202	202	202	202	202	202	202	202	202	2	2
YA00245993	Human	Blood culture	9	Male	2020	3	28	Africa	South Africa	Western Cape		PRJEB39546	SAMEA9966845	263900	260211	260211	202	202	202	202	202	202	202	202	202	2	2
YA00245995	Human	Blood culture	28	Female	2021	3	24	Africa	South Africa	Western Cape		PRJEB39546	SAMEA9966844	280993	280993	280993	154379	154379	202	202	202	202	202	202	202	2	2
YA00248238	Human	Stool	9	Male	2021	4	3	Africa	South Africa	Western Cape		PRJEB39546	SAMEA10113739	263900	260211	260211	202	202	202	202	202	202	202	202	202	2	2
YA00252219	Human	Stool	7	Female	2021	4	19	Africa	South Africa	Western Cape	Cluster 2	PRJEB39546	SAMEA9966453	270313	270313	256191	202	202	202	202	202	202	202	202	202	2	2
YA00262313	Human	Blood culture	11	Male	2021	5	16	Africa	South Africa	Western Cape	Cluster 2	PRJEB39546	SAMEA9967205	283881	283881	256191	202	202	202	202	202	202	202	202	202	2	2
YA00264263	Human	Blood culture	33	Female	2021	5	25	Africa	South Africa	Western Cape	Cluster 3	PRJEB39546	SAMEA9966563	275476	275476	12503	202	202	202	202	202	202	202	202	202	2	2
YA00266072	Human	Blood culture	33	Male	2021	6	3	Africa	South Africa	Western Cape	Cluster 3	PRJEB39546	SAMEA9966843	280992	275476	12503	202	202	202	202	202	202	202	202	202	2	2
YA00266090	Human	Blood culture	10	Male	2021	6	3	Africa	South Africa	Western Cape	Cluster 3	PRJEB39546	SAMEA9966924	281401	275476	12503	202	202	202	202	202	202	202	202	202	2	2
YA00267250	Human	Blood culture	11	Female	2021	5	20	Africa	South Africa	Western Cape	Cluster 1	PRJEB39546	SAMEA9966923	281400	249364	26478	26478	202	202	202	202	202	202	202	202	2	2
YA00268135	Human	Blood culture	18	Female	2021	6	6	Africa	South Africa	Western Cape	Cluster 3	PRJEB39546	SAMEA9966841	280921	280921	12503	202	202	202	202	202	202	202	202	202	2	2
YA00268147	Human	Blood culture	38	Male	2021	6	6	Africa	South Africa	Western Cape		PRJEB39546	SAMEA9966840	280922	280922	280922	280922	280922	280922	12514	202	202	202	202	202	2	2
YA00271226	Human	Blood culture	15	Male	2021	6	22	Africa	South Africa	Western Cape		PRJEB39546	SAMEA9966875	281198	281198	281198	202	202	202	202	202	202	202	202	202	2	2
YA00274212	Human	Blood culture	11	Male	2021	7	11	Africa	South Africa	Western Cape	Cluster 2	PRJEB39546	SAMEA9966838	280918	280918	256191	202	202	202	202	202	202	202	202	202	2	2
YA00274673	Human	Blood culture	2	Male	2021	7	11	Africa	South Africa	Western Cape		PRJEB39546	SAMEA9966848	281156	281156	107872	202	202	202	202	202	202	202	202	202	2	2
YA00276175	Human	Blood culture	8	Female	2021	7	3	Africa	South Africa	Western Cape	Cluster 3	PRJEB39546	SAMEA9966851	281159	275476	12503	202	202	202	202	202	202	202	202	202	2	2
YA00276545	Human	Blood culture	38	Male	2021	7	25	Africa	South Africa	Western Cape	Cluster 3	PRJEB39546	SAMEA9967117	281159	275476	12503	202	202	202	202	202	202	202	202	202	2	2
YA00277547	Human	Blood culture	26	Female	2021	8	1	Africa	South Africa	Western Cape		PRJEB39546	SAMEA13259909	297379	221026	208099	202	202	202	202	202	202	202	202	202	2	2
YA00279014	Human	Blood culture	24	Female	2021	8	10	Africa	South Africa	Western Cape	Cluster 3	PRJEB39546	SAMEA10558566	285244	285244	221038	202	202	202	202	202	202	202	202	202	2	2
YA00279017	Human	Blood culture	16	Male	2021	8	12	Africa	South Africa	Western Cape	Cluster 3	PRJEB39546	SAMEA110107597	309426	275476	12503	202	202	202	202	202	202	202	202	202	2	2
YA00280328	Human	Blood culture	21	Female	2021	8	25	Africa	South Africa	Western Cape	Cluster 2	PRJEB39546	SAMEA10558642	285139	285139	256191	202	202	202	202	202	202	202	202	202	2	2
YA00280639	Human	Blood culture	27	Male	2021	8	29	Africa	South Africa	Western Cape	Cluster 2	PRJEB39546	SAMEA10558558	285114	270313	256191	202	202	202	202	202	202	202	202	202	2	2
YA00282946	Human	Blood culture	54	Male	2021	9	19	Africa	South Africa	Western Cape	Cluster 3	PRJEB39546	SAMEA13259583	281159	275476	12503	202	202	202	202	202	202	202	202	202	2	2
YA00283872	Human	Blood culture	47	Female	2021	9	27	Africa	South Africa	Western Cape		PRJEB39546	SAMEA13259584	291156	291156	26669	202	202	202	202	202	202	202	202	202	2	2
YA00283879	Human	Blood culture	5	Female	2021	9	28	Africa	South Africa	Western Cape	Cluster 2	PRJEB39546	SAMEA13423296	291745	291745	256191	202	202	202	202	202	202	202	202	202	2	2
YA00284670	Human	Blood culture	7	Female	2021	9	26	Africa	South Africa	Western Cape	Cluster 2	PRJEB39546	SAMEA13423219	280918	280918	256191	202	202	202	202	202	202	202	202	202	2	2
YA00285211	Human	Blood culture	29	Female	2021	10	9	Africa	South Africa	Western Cape		PRJEB39546	SAMEA13423188	291156	291156	26669	202	202	202	202	202	202	202	202	202	2	2
YA00287798	Human	Stool	1	Male	2021	10	25	Africa	South Africa	Western Cape	Cluster 2	PRJEB39546	SAMEA13259666	292995	292995	256191	202	202	202	202	202	202	202	202	202	2	2
YA00289513	Human	Blood culture	29	Male	2021	11	15	Africa	South Africa	Western Cape	Cluster 3	PRJEB39546	SAMEA13259706	295415	295415	12503	202	202	202	202	202	202	202	202	202	2	2
YA00289515	Human	Blood culture	1	Male	2021	11	14	Africa	South Africa	Western Cape		PRJEB39546	SAMEA13259679	295417	295417	295417	202	202	202	202	202	202	202	202	202	2	2
YA00290821	Human	Blood culture	11	Male	2021	11	15	Africa	South Africa	Western Cape	Cluster 2	PRJEB39546	SAMEA13259760	295926	295926	256191	202	202	202	202	202	202	202	202	202	2	2
XB01311126	Human	Blood culture	8	Male	2021	11	28	Africa	South Africa	Western Cape	Cluster 2	PRJEB39546	SAMEA13423388	299606	299606	256191	202	202	202	202	202	202	202	202	202	2	2
YA00292095	Human	Blood culture	31	Male	2021	12	3	Africa	South Africa	Western Cape	Cluster 3	PRJEB39546	SAMEA13259673	295399	295399	12503	202	202	202	202	202	202	202	202	202	2	2
YA00293842	Human	Stool	5	Female	2021	12	3	Africa	South Africa	Western Cape	Cluster 2	PRJEB39546	SAMEA13259763	295929	295929	295929	202	202	202	202	202	202	202	202	202	2	2
YA00293847	Human	Blood culture	51	Male	2021	12	10	Africa	South Africa	Western Cape		PRJEB39546	SAMEA13259765	295930	295930	107872	202	202	202	202	202	202	202	202	202	2	2
YA00293855	Human	Blood culture	12	Male	2021	12	8	Africa	South Africa	Western Cape	Cluster 2	PRJEB39546	SAMEA13259761	295927	295927	256191	202	202	202	202	202	202	202	202	202	2	2
YA00293859	Human	Blood culture	15	Male	2021	12	10	Africa	South Africa	Western Cape	Cluster 3	PRJEB39546	SAMEA13259762	295928	295415	12503	202	202	202	202	202	202	202	202	202	2	2
YA00294684	Human	Blood culture	5	Male	2021	12	26	Africa	South Africa	Western Cape		PRJEB39546	SAMEA13259962	295930	295930	107872	202	202	202	202	202	202	202	202	202	2	2
